# Highlighting the Influence between Physical and Chemical Cross-Linking in Dynamic Hydrogels for Two-Photon Micropatterning

**DOI:** 10.1021/acs.biomac.5c00062

**Published:** 2025-05-09

**Authors:** Antonella Fantoni, Alice Salvadori, Aleksandr Ovsianikov, Robert Liska, Stefan Baudis

**Affiliations:** † Institute of Applied Synthetic Chemistry, Technische Universität Wien, Getreidemarkt 9, 1060 Vienna, Austria; ‡ Austrian Cluster for Tissue Regeneration, 1200 Vienna, Austria; § Institute of Materials Science and Technology, Technische Universität Wien, Getreidemarkt 9, 1060 Vienna, Austria; ∥ Christian Doppler Laboratory for Advanced Polymers for Biomaterials and 3D Printing, Getreidemarkt 9, 1060 Vienna, Austria

## Abstract

Photolabile hydrogels have gained tremendous interest for a wide range of applications in materials and life sciences. Usually, photodegradability is introduced via chromophores and labile bonds, making such materials intrinsically light sensitive. In recent years, disulfide bonds have emerged as an innovative alternative, as they can be selectively cleaved in the presence of (photo)­generated radicals. However, such materials suffer from limited network stability and high swelling as a result of thiol–disulfide metathesis reactions. Herein, we present two strategies to counteract such phenomena by network stabilization either via physical or chemical incorporation of (un)­modified gelatin macromers to norbornene-modified poly­(vinyl alcohol) networks. Photolabile behavior was introduced by a simple disulfide-containing dithiol cross-linker. Tunable material properties were investigated by means of in situ photorheology, in vitro swelling, and degradation experiments. Finally, we demonstrate an innovative method for localized disulfide cleavage via two-photon micropatterning.

## Graphical abstract

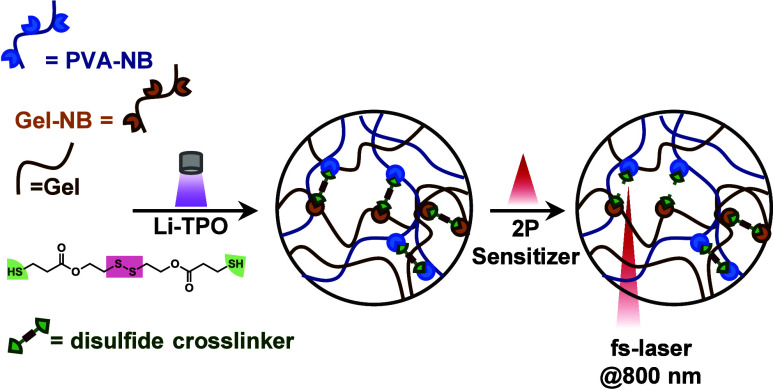

## Introduction

Hydrogels represent highly hydrated polymers that are able to swell and thus retain water as a result of physical or chemical cross-linking within the gel.^1^ Lately, evermore interest has shifted toward photoresponsive hydrogels, allowing for local tuning of the physicochemical properties of a material by irradiation with light.^2^ Thereby, a variety of responses can be triggered, including secondary gel formation^3^ or degradation,^4,5^ network contraction or expansion,^6^ as well as uncaging of target molecules.^7^ To facilitate such light-controlled reactions, a great toolbox of photoresponsive chemistries has been developed, including *o-*nitrobenzyl groups,^8^ coumarin derivatives,^9^ or ruthenium complexes.^10^ In brief, all of these phototriggers comprise chromophores, allowing light absorption and labile bonds that cleave upon irradiation. However, the inherent light sensitivity and usually multistep, complex synthesis have limited the widespread application of such materials up to now.

An elegant method to overcome these issues is the introduction of disulfide bonds that can be cleaved by photogenerated radicals^11^ but are insensitive to degradation under ambient light.^12^ Although disulfides are susceptible to photocleavage at high-intensity UV light, the presence of a radical-generating species, e.g., a radical photoinitiator, cleaves the disulfide bonds via a radical-mediated fragmentation reaction.^11^ In detail, radicals attack the disulfide bond, producing thiyl radicals that are able to undergo fragmentation or exchange reactions, enabling the material to become dynamic via thiol–disulfide metathesis. Frequently, disulfide functionalities are directly introduced via oxidative coupling of thiols to disulfides,^11,13,14^ entailing the disadvantages of long reaction times and challenging reaction handling. Thus, the design of disulfide linkers with polymerizable end-groups represents a striking alternative. A variety of acrylate-terminated disulfide linkers have been studied and were either incorporated via free radical copolymerization with acyrlates^15,16^ or thiol-Michael additions.^17^ Recently, light-initiated thiol–ene reactions involving norbornene-terminated disulfide linkers were reported.^5^ Network formation was favored at low concentrations of photogenerated radicals, while photoscission of the labile bonds was solely permitted via two-photon (2P) micropatterning involving pulsed IR lasers and 2P-absorbing radical sources. Another example of wavelength-orthogonal disulfide photoscission was presented by Alfarhan et al.^18^ Thiol-terminated disulfide cross-linkers were photopolymerized with acrylated monomers with green light (500–700 nm) under photobase catalysis, while radical-mediated photodegradation occurred only with blue (329–500 nm) light.

Interestingly, extensive and uncontrollable swelling of disulfide-containing hydrogels has been reported, resulting from thiol–disulfide metathesis, leading to an expansion of the network.^5,11^ This volumetric increase can pose a problem in geometrically demanding applications, e.g., microfluidic chips, leading to a decrease in mechanical performance and deformation of the desired structure.^19^ Herein, we present the use of simple disulfide-containing dithiol cross-linkers for biocompatible norbornene-modified poly­(vinyl alcohol) (PVA-NB) to prepare form-stable hydrogels either via physical interactions with unmodified gelatin or chemical incorporation of norbornene-modified gelatin (Gel-NB) (Scheme 1). Network formation via fast thiol–ene photo-cross-linking at low radical PI concentrations in the one-photon (1P) case was characterized via in situ photorheology, and the influence of physical or chemical interactions between the macromer subnetworks was assessed by swelling and in vitro degradation studies. Finally, this study highlights the applicability of photoresponsive hydrogels for two-photon micropatterning, which further expands the utilization of disulfide-containing hydrogels.

**Scheme 1:**
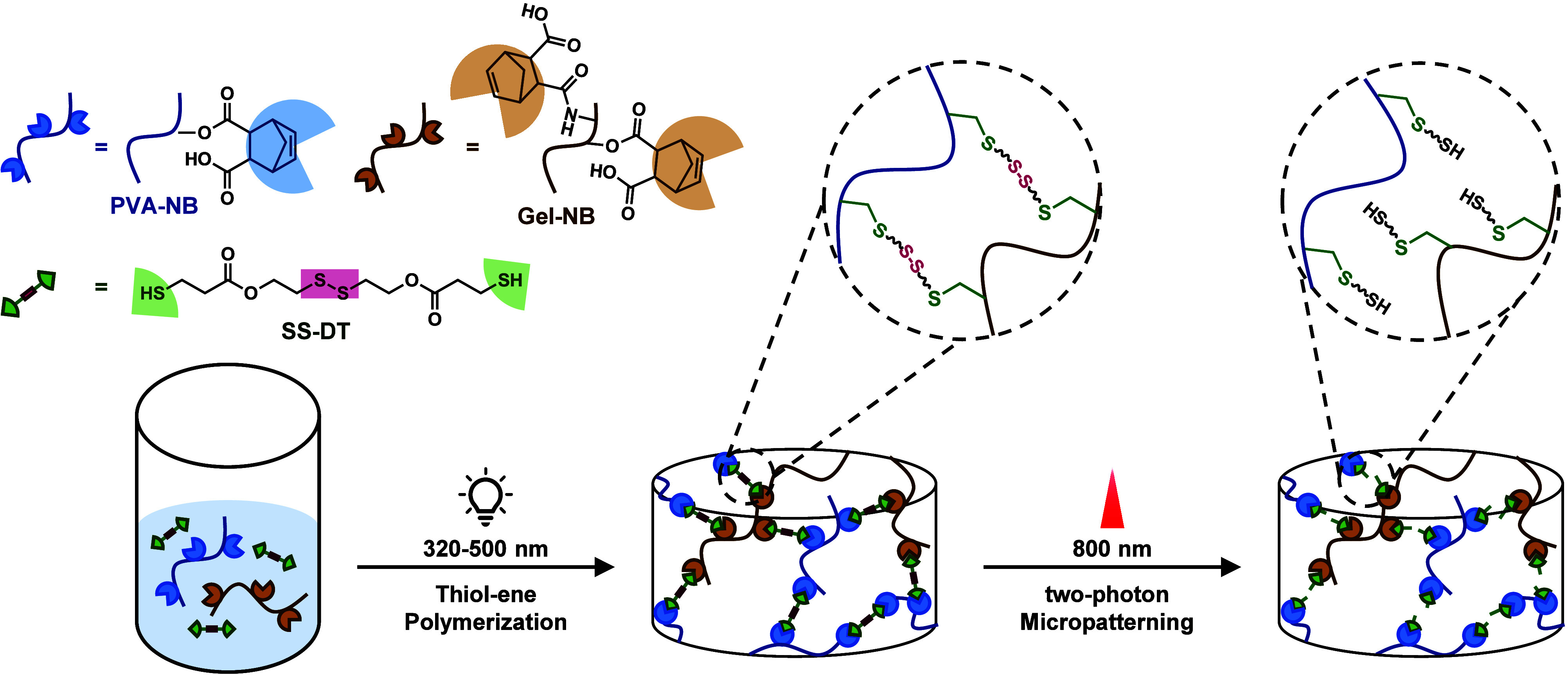
Orthogonal Hydrogel Formation and Degradation by Combining Thiol–Ene Polymerization (Left) with Two-Photon-Mediated Disulfide Scission (Right)

## Experimental Section

### Materials

Poly­(vinyl alcohol) (*M*_w_ 27 kDa, 98% degree of hydrolysis), gelatin (type A, bloom 300 from porcine skin), and bis­(2-hydroxyethyl)­disulfide were purchased from Sigma-Aldrich. *cis*-5-Norbornene-*endo*-2,3-dicarboxylic anhydride (carbic anhydride) was obtained from Acros. *p*-Toluenesulfonic acid (*p*-TsOH) and 3-mercaptopropionic acid were acquired from TCI Europe. Dimethyl sulfoxide (DMSO) was obtained from Panreac AppliChem. Commercial grade toluene (Donau Chemie) was dried using a PureSolv system (Inert, Amesbury, MA). The photoinitiator lithium phenyl-2,4,6-trimethylbenzoylphosphinate (Li-TPO) and the photosensitizer sodium 3,3′-((((1E,1′E)-(2-oxocyclopentane-1,3-diylidene)­bis­(methanylylidene))­bis­(4,1-phenylene))­bis­(methylazanediyl)) dipropanoate (P2CK) were synthesized at TU Wien following literature procedures.^20,21^

### Instrumentation

Column chromatography was performed on a Büchi Sepacore flash system (Büchi pump module C-605, Büchi control unit C-620, Büchi UV-photometer C-635, Büchi fraction collector C-660) using glass columns packed with silica gel (Merck).

NMR spectra were recorded on a Bruker Avance spectrometer at 400 MHz for ^1^H and 100 MHz for ^13^C. Spectra of photosensitive compounds were exclusively measured in brown-glass NMR tubes. Data for ^1^H NMR are reported as follows: chemical shift (δ) in units of parts per million (ppm) from tetramethylsilane (TMS) using the residual nondeuterated solvent signal of CD_2_Cl_2_ (δ = 5.32 ppm), CDCl_3_ (δ = 7.26 ppm), D_2_O (δ = 4.79 ppm), or DMSO-*d*_6_ (δ = 2.50 ppm) as an internal reference. Multiplicities are reported by using the following abbreviations—bs: broad singlet; s: singlet; d: doublet; t: triplet; m: multiplet. ^13^C NMR data are reported in ppm from TMS using the central peak of the solvent as a reference (CD_2_Cl_2_: δ = 53.84 ppm, CDCl_3_: δ = 77.16 ppm, DMSO-*d*_6_: δ = 39.52 ppm).

### Synthesis of PVA-NB

Norbornene-modified poly­(vinyl alcohol) (PVA-NB) was synthesized similarly based on a study by Baudis et al.^22^ In brief, PVA (10.0 g, 218 mmol) was dissolved in 66 mL of DMSO and stirred in vacuo (44 mbar) at 70 °C for 3 h. Thereafter, the reaction apparatus was purged with argon, and *p-*TsOH (20.3 mg, 0.118 mmol) and carbic anhydride (2.87 g, 17.4 mmol), diluted with 27 mL of dry DMSO, were added to the colorless solution over 20 min. The resulting solution was stirred at 60 °C for 18 h. The reaction mixture was dialyzed (Spectrum Labs MWCO 12–14 kDa) against 10 mM sodium bicarbonate solution (4 h) and thereafter against deionized water for 48 h with frequent medium changes (∼2 h). After dialysis, the aqueous solution was concentrated in vacuo and lyophilized (−85 °C, 0.01 mbar, Christ Gamma 2–20 freeze dryer) to give the desired product in 97% yield as a white powder. The characteristic peaks of norbornene (6.3–6.2 ppm) were compared with the reference −CH– peaks of PVA (3.9–4.0 ppm) to give a degree of substitution (DS) of 8% (Figure S1).

### Synthesis of Gel-NB

Norbornene-modified gelatin (Gel-NB) was prepared based on a modified procedure by Munoz et al.^23^ Herein, 5.00 g of gelatin (type A, bloom 300, porcine skin, ∼1.7 mmol amino groups) was dissolved in 50 mL of anhydrous DMSO at 60 °C. Once gelatin was dissolved, the temperature was lowered to 50 °C, and carbic anhydride (2.51 g, 15.2 mmol) was added as a solid. The resulting solution was stirred at 50 °C for 16 h. The reaction mixture was diluted with 30 mL of mΩ water and dialyzed against 10 mM sodium bicarbonate solution (4 h), 200 mM NaCl solution (12 h), and thereafter against mΩ water for 48 h. All dialysis steps were conducted at 40 °C to prevent physical gelation of Gel-NB. The molecular weight cutoff of the dialysis tubes was 12–14 kDa (Spectrum Labs). After dialysis, the pH value of the solution was adjusted to 7.4 by the addition of a 0.1 M NaOH solution. The aqueous solution was concentrated in vacuo and lyophilized (−85 °C, 0.01 mbar, Christ Gamma 2–20 freeze dryer) to give the desired product in 80% yield as an off-white solid. The product was characterized via ^1^H NMR spectroscopy using TMSP (3-(trimethylsilyl)­propionic-2,2,3,3-d_4_ acid sodium salt) as an internal standard.^24^ The resulting DS (the number of modification moieties per gelatin modification weight) was determined to be 0.217 mmol/g (Figure S2).

### Synthesis of SS-DT

The synthesis was performed using a modified procedure by Li et al.^25^ The reaction was conducted under an argon atmosphere using a Dean–Stark apparatus. Bis­(2-hydroxyethyl) disulfide (2.50 g, 16.2 mmol, 1 equiv), 3-mercaptopropionic acid (3.45 g, 32.4 mmol, 2 equiv), and *p-*TsOH (90.2 mg, 0.49 mmol) were dissolved in 18 mL of toluene. The solution was refluxed for 18 h, and the progress of the reaction was followed via ^1^H NMR. After cooling to room temperature, the colorless solution was washed with sat. aq. NaHCO_3_ (2 × 30 mL), water (2 × 30 mL), and brine (30 mL). Pooled aqueous phases were washed with toluene (3 × 50 mL). Combined organic layers were dried over Na_2_SO_4_, and the solvent was removed in vacuo. The crude oil was purified by column chromatography (silica gel, PE:EE = 3:1) to give a colorless oil in 81% yield.

^1^H NMR (400 MHz, CDCl_3_): δ 4.37 (t, *J* = 6.5 Hz, 4H), 2.93 (t, *J* = 6.5 Hz, 4H), 2.81–2.74 (m, 4H), 2.67 (td, *J* = 6.6, 1.1 Hz, 4H), 1.66 (t, *J* = 8.3 Hz, 2H) (Figure S3).

^13^C NMR (100 MHz, CDCl3): δ 171.5, 62.6, 38.5, 37.3, 19.8 (Figure S4).

### Preparation of Hydrogel Formulations

First, stock solutions of the photoinitiator (PI) Li-TPO (0.6–17 mM) in phosphate-buffered saline (PBS, pH 7.4) were prepared. Thereafter, the respective amounts of macromers (PVA-NB, Gel-NB) were dissolved in the PI stock solution. The cross-linker SS-DT was dissolved in DMSO/H_2_O = 70:30 (v%) to achieve a concentration of 0.1 mg mL^–1^. For semi-IPNs, unmodified gelatin was dissolved in PBS to obtain a stock solution of 20 wt %. Afterward, 100 μL of the PVA-NB (10–20 wt %) solutions was added to 100 μL of the gelatin solution and homogenized at 37 °C. Thereafter, SS-DT was added (0.1 mg mL^–1^ in 70/30 v% DMSO/H_2_O). An equimolar thiol/ene ratio was used herein. Thereby, the final concentration of macromers and gelatin was halved, giving prepolymer formulations of 5–10 wt % PVA-NB and 10 wt % gelatin. The semi-IPNs were classified according to their PVA-NB content (IPN5, IPN7.5, and IPN10).

For PVA-NB/Gel-NB hybrid gels, 100 μL of PVA-NB (10–20 wt %) and 100 μL of Gel-NB (20 wt %) stock solutions were homogenized at 37 °C, and SS-DT was added thereafter (0.1 mg mL^–1^ in 70/30 v% DMSO/H_2_O). Thereby, the final concentration of the macromers was 10 wt % Gel-NB + 5–10 wt % PVA-NB, keeping an equimolar ratio of thiol and norbornene groups.

### Photorheological Analysis

Rheological measurements were performed on an Anton-Paar Modular Compact Rheometer MCR 302 WESP with a disposable plate-to-plate measuring system (PP08 disposable, Ø = 8 mm; gap size = 0.5 mm). All measurements were performed in oscillation mode with a strain of 0.2% and at a frequency of 1 Hz. As a light source, an Omnicure lamp with filtered UV light (320–500 nm) was used. The light intensity was measured directly on top of the glass slide with a USB2000+ radiometer from OceanOptics. For each measurement, 28 μL of the formulation was used. The precursor formulations were protected from water loss through evaporation by adding a paraffin ring around the sample. Measurements started with a blank period of 60 s (37 °C) in oscillation mode without UV light. For neat PVA-NB:SS-DT measurements, the UV light was switched on with a light intensity of 20 mW cm^–2^. The samples were irradiated for 600 s. For semi*-*IPNs and hybrid gels, the blank period was followed by a decrease in the temperature to 21 °C over 5 min. To enable physical cross-linking of the gelatin, the temperature was kept at 21 °C for 20 min. Afterward, the UV light was switched on with a light intensity of 20 mW cm^–2^. The samples were irradiated for 600 s. All measurements were conducted in triplicate. The produced hydrogels were utilized for subsequent swelling tests in PBS.

### Hydrogel Swelling Experiments

The polymer discs, obtained during photorheology, were removed from the photorheometer stamp and placed in individual tared glass vials, and the hydrogels were swollen in 2 mL of PBS for 24 h. Thereafter, the swollen gels were gently patted dry with a paper towel to remove surface water, and the swollen weight (*m*_swollen_) was determined. After lyophilization (−85 °C, 0.01 mbar, Christ Gamma 2–20 freeze dryer), the dry weight (*m*_dry_) was determined, and the mass swelling ratio (MSR) was determined according to eq 1.^26^

$$\mathrm{Mass}\;\mathrm{swelling}\;\mathrm{ratio}\;(\mathrm{MSR})=\frac{m_{\mathrm{swollen}}}{m_{\mathrm{dry}}}$$ 1

### In Vitro Degradation Study

Hydrogel formulations were cured in transparent silicon molds with a glass lid to prevent the evaporation of water. Cylindrical-shaped specimens with dimensions of *d* = 10 and *h* = 5 mm were preheated to 37 °C and filled with ∼150 μL of formulation. Thereafter, the molds were cooled to 21 °C for 20 min and subsequently photopolymerized using a Lumamat 100 light oven with six Osram Lux L Blue 18 W lamps (10 min, 400–580 nm, ∼20 mW cm^–2^). Degradation studies were conducted in PBS (pH 7.4), prepared as reported in the literature.^27^ Each hydrogel class was tested in triplicate. The initial mass (*m*_0_) was determined with a balance of 0.01 mg precision, and the hydrogels were afterward immersed in 20 mL of buffer medium and stored in a climate chamber at 37 °C. After 1, 2, 7, 14, 21, 30, 60, 75, and 90 days, three samples of each hydrogel category were removed and reconditioned for 30 min in deionized water, and the surface was gently dried with an absorbent wipe and weighed directly (*m*_swollen_) to determine the swelling according to eq 1. Reconditioned samples were frozen (−30 °C) and lyophilized, and the dry weight (*m*_dry_) was determined to calculate the mass loss (% initial weight) of each time point (*t* = *x* days) following eq 2:

$$\%\mathrm{initial}\;\mathrm{mass}=\frac{m_{\mathrm{dry},t_{x}}}{m_{\mathrm{dry},t_{0}}}\times 100$$ 2

At regular time intervals, the pH value of the buffer solutions was checked to ensure constant degradation conditions.

### Two-Photon Micropatterning

Prepolymer formulations (50 μL) consisting of 5 wt % PVA-NB:SS:DT and either 10 wt % Gel or Gel-NB:SS-DT containing 0.6 mM Li-TPO as PI in PBS were polymerized in square silicon molds (4 × 4 mm^2^, *d* = 3 mm) between methacrylated glass-bottom μ-dishes (35 mm, Ibidi GmbH) and cover glass in a Lumamat 100 light oven with six Osram Lux L Blue 18 W lamps (10 min, 400–580 nm, ∼20 mW cm^–2^). After the removal of the silicon molds, the solidified hydrogel disks were swollen in PBS for 18 h. Subsequently, the hydrogels were submersed in solutions of P2CK in PBS at concentrations of 0.5–2.0 mM for at least 4 h to allow diffusion of the chromophore into the network. Afterward, samples were cut using a scalpel to generate a sharp edge. Micropatterning was performed by means of two-photon degradation. Details of a modified setup have been published recently.^4^ Briefly, the experimental setup consists of a tunable femtosecond near-infrared (NIR) laser (MaiTai eHP DeepSee, Spectra-Physics), with a pulse duration of 70 fs, a repetition rate of 80 MHz, and equipped with a 10*x*/0.4 (Olympus, Tokyo, Japan) objective. The micropatterning was performed at 800 nm. In particular, parallel channels with a length of 700 μm and a cross-section of 30 × 50 μm^2^ were eroded from the edge into the bulk hydrogel at a height of 150 μm above the glass bottom with varied laser powers (10–150 mW, Δ*P* = 10 mW) and scanning speeds (200–600 mm s^–1^, Δ*v* = 100 mm s^–1^). A line spacing (*D*_*xy*_) of 0.2 μm and a layer height (*D*_*z*_) of 0.5 μm were chosen. After micropatterning, the hydrogels were washed with PBS twice and submersed in a solution of fluorescent, high-molecular-weight FITC-dextran (FITC2000, 1 mg mL^–1^, *M*_w_ ∼ 2000 kDa) in PBS at room temperature for 2 h. Channels were visualized by laser scanning microscopy (LSM800, ZEISS) 18 h and 7 days after fabrication. The channel’s quality was assessed by measuring the channel width and fluorescence inside the channels (normalized to the fluorescence signal in the surrounding material). Therefore, the channels were divided into three equal segments, and measurements were conducted therein.

## Results and Discussion

### Design of Photopolymerizable Macromers and Cross-Linkers

The hydrogels in this study are composed of a disulfide-containing dithiol cross-linker (SS-DT) and high-molecular-weight macromers equipped with norbornene functional groups. Generally, thiol–norbornene photopolymerization is known to combine exceptionally fast cross-linking, spatiotemporal control during network formation, and the ability to be conducted under physiological conditions at low photoinitiator concentrations.^28^ Another important feature for the desired disulfide-bearing hydrogels is the orthogonality of the thiol–norbornene conjugation, as a strictly alternating coupling of both thiol and norbornene groups prevails during network formation.

Herein, poly­(vinyl alcohol) (PVA) was chosen as the primary macromer, as it possesses countless possibilities for the modification of its secondary hydroxyl groups.^22^ Thus, norbornene-functionalized PVA (PVA-NB, Scheme 2) was synthesized in a degree of substitution (DS) of 8% (Figure S1). Due to the high molecular weight of the precursor (*M*_w_ 27 kDa), thiol-cross-linking of PVA-NB results in comparably stiff hydrogels. Thus, we envisioned that the addition of a second, “softer” network would allow us to easily adjust the overall material properties by varying the amounts of stiff and soft components.

**Scheme 2:**
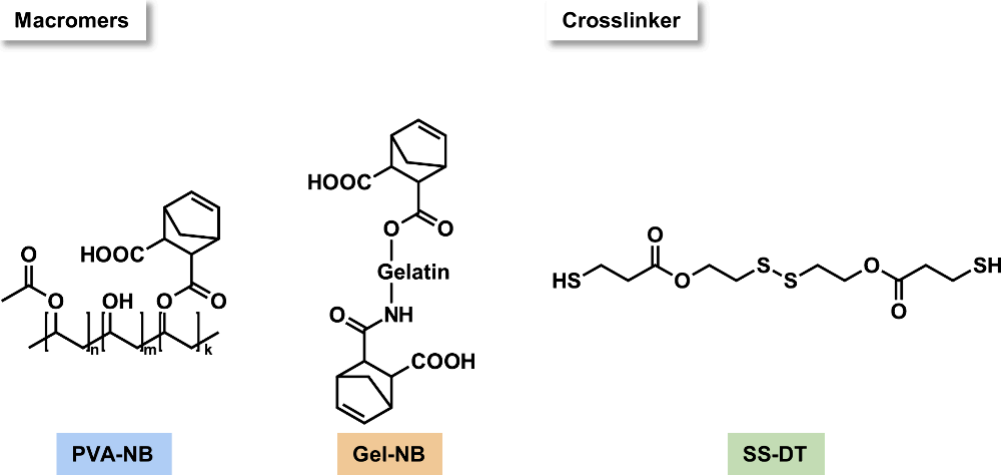
Norbornene-Functionalized Macromers PVA-NB and Gel-NB and the Disulfide-Bearing Cross-Linker SS-DT

Gelatin, a naturally occurring denatured collagen, has gained increased attention in hydrogel fabrication. The first strategy of gelatin incorporation utilizes the sol–gel transition when the gelatin solution is below a temperature-dependent critical value.^28^ Consequently, gelatin forms triple helices caused by strong hydrogen bonds between the polypeptide chains.^29^ Thus, combining thiol–ene cross-linked PVA networks with physically intertwined gelatin (Gel), a semi-Interpenetrating Polymer Network (semi-IPN), will be formed.

Additionally, the abundant number of both amine and hydroxyl groups on the gelatin backbone permits simple modification with photopolymerizable groups. Thus, norbornene-modified gelatin (Gel-NB, Scheme 2) was prepared in a low DS (0.217 mmol g^–1^, Figure S2). As the ability for physical cross-linking of modified gelatins decreases with increasing DS,^29^ a low DS was aimed for. This pregelation state is known to be beneficial for later covalent cross-linking steps, as it prematurely accumulates the functional groups before the photopolymerization.^30^ By simultaneous thiol–ene polymerization of PVA-NB/Gel-NB and SS-DT, hybrid hydrogels will be obtained. For both concepts (semi-IPNs and hybrid gels), physical cross-linking between the subnetworks should furthermore stabilize the disulfide-containing networks and reduce the problem of uncontrollable swelling.

### Optimizing the Thiol–Ene Photopolymerization

Before the in-depth analysis of semi-IPNs and hybrid hydrogels was conducted, the optimal conditions for the thiol–ene conjugation were elucidated. Therefore, the simple model reaction consisting of PVA-NB as the norbornene-bearing macromer, SS-DT as the cross-linker, and Li-TPO as the radical photoinitiator (PI) was investigated by in situ photorheology. Rheological analysis was performed to analyze the network formation with varying amounts of PI and macromer content. Thereby, a limiting concentration of Li-TPO should be established, as an excess of the PI induces radical-mediated degradation of the disulfide cross-linked hydrogels.^5,11^ Simultaneously, in situ photorheology allows for the joint analysis of photoreactivity and mechanical properties of the materials.^31^ Herein, 5–10 wt % of PVA-NB was reacted with an equimolar amount (in respect to functional groups) of the cross-linker SS-DT by irradiation with filtered UV light (320–500 nm, 20 mW cm^–2^, Figure 1A and Table S1). After 60 s of equilibration, the prepolymer solutions were irradiated for 300–600 s.

**Figure 1:**
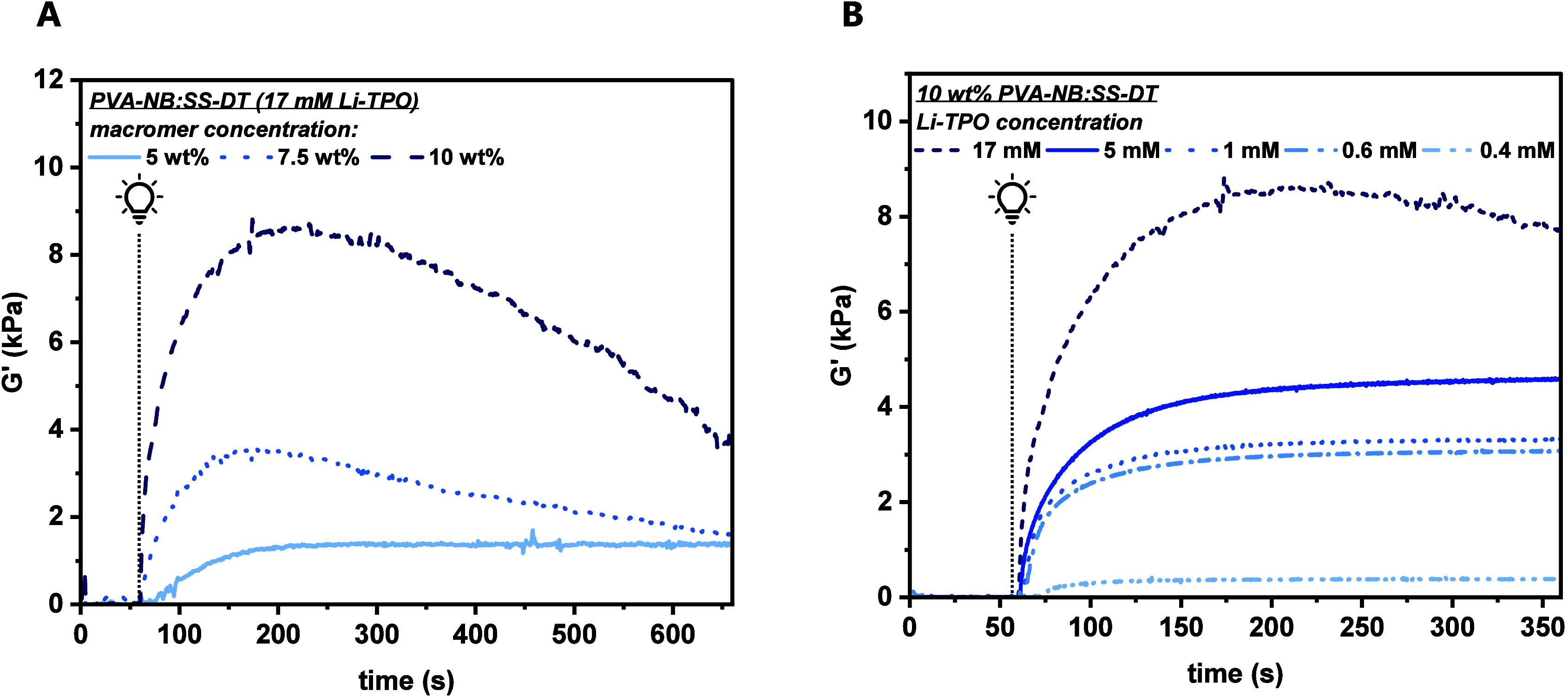
In situ photorheology measurements containing (A) 5–10 wt % PVA-NB and 17 mM Li-TPO as PI and (B) 10 wt % PVA-NB with varying amounts of Li-TPO (0.4–17 mM). An equimolar amount of SS-DT was used as the cross-linker. Irradiation started at 65 s (marked with a light bulb).

At high PI concentrations (17 mM, Figure 1A) a rapid increase in the storage modulus (*G*′) was observed within the first 60 s of irradiation, stemming from the rapid thiol–norbornene cross-linking. Thereby, the maximum *G*′ varied from 9.8 kPa (10 wt %) to 4.0 kPa (7.5 wt %) and 1.5 kPa (5 wt %). However, a subsequent decrease in *G′* for 7.5–10 wt % PVA-NB:SS-DT indicates network degradation as a result of the excess of radicals present in the model system. Thus, the thiol–ene conjugation (TEC) is favored over radical-mediated disulfide cleavage in the beginning. Similar observations have been made by Bowman et al., where it has been found that thiol–ene reactions form a (∼30 times) faster first-stage matrix before subsequent disulfide–ene reactions occur.^32,33^ Thus, the fast thiol–ene reaction will also dominate in the early stages of the presented hydrogel system. However, it has to be considered that disulfide–ene reactions will, of course, alter the formed network. This phenomenon seems to be especially present in hydrogels produced from the 5 wt % prepolymer solution as the *G′* plateau was reached without any detectable material scission. Two possible scenarios may induce this phenomenon. First, the concentration of cleavable moieties could simply be too low in the produced gel. Second, disulfide–ene reactions could interfere with the thiol–ene reactions, resulting in a decelerated cross-linking reaction.

To suppress network degradation, the PI concentration was varied from 17 to 0.4 mM in a 10 wt % PVA-NB prepolymer formulation (Figure 1B and Table S2). For the lowest Li-TPO concentration (0.4 mM), a significant delay (>10 s) in TEC was observed. By increasing the PI concentration, delay times of ∼3 s (0.6–1 mM) or even below 1 s (5–17 mM) were obtained. Interestingly, no substantial network degradation was observed at PI concentrations ≤5 mM. However, the final *G′* decreased from 4.7 kPa (5 mM) to 3.4 kPa (0.6 mM). Reducing the photoinitiator concentration is known to influence not only the reactivity but also the final cross-linking density and, thus, storage moduli.^34,35^ Resulting from this model study, a Li-TPO concentration of 0.6 mM was chosen, as it suppresses network degradation without forfeiting too much gel stiffness.

### Preparation and Characterization of Semi-IPNs Containing PVA-NB:SS-DT and Gelatin

Recently, Lunzer et al. showed that disulfide-based hydrogels suffer from uncontrollable swelling after preparation via TEC.^5^ Therefore, we chose to add a second, non-photodegradable, physical network that interacts with the TEC hydrogel via strong hydrogen bonds. It is well-known that gelatin (Gel) can be incorporated into PVA-based scaffolds via strong intermolecular H-bonds.^36–38^ However, to the best of our knowledge, no prior work has examined the addition of a photounresponsive network to a photodegradable PVA scaffold.

At first, the network formation and photoreactivity were assessed by in situ photorheological analysis. Similar to the model study, the PVA-NB:SS-DT concentration varied from 5 to 10 wt %, whereas the gelatin content was consistent in all formulations with 10 wt %. For ease of understanding, materials will now be termed IPN5, IPN7.5, or IPN10, referring to the PVA-NB:SS-DT content of each semi-IPN. Furthermore, the established Li-TPO concentration of 0.6 mM was used. As the underlying hydrogel system contains not only the chemical photo-cross-linking but also the physical gelation of gelatin, a modified procedure, as presented by Rebers et al., was used herein (Figure S6).^29^ In brief, the physical gelation of gelatin was induced by cooling from 37 to 21 °C within 5 min, followed by isothermal treatment at 21 °C for 20 min. Only thereafter, the photoconjugation of PVA-NB:SS-DT was induced. It is well-known that the macromer concentration directly influences the network density and photoreactivity due to the amount of available cross-linking moieties. Indeed, the final *G′* increased from IPN5 (5.0 kPa) to IPN10 (7.0 kPa), accompanied by shorter delay times (IPN5:3.6, IPN10:2.9 s) with higher macromer content (Figure 2A and Table S4). Thus, gel formation was observed for all tested semi-IPNs at the previously established low Li-TPO concentration of 0.6 mM, and no radical-mediated disulfide scission was observed. These results support the previous findings that gel formation via thiol–norbornene cross-linking is favored over photoerosion. Interestingly, the physical cross-linking of gelatin was influenced by the residual dissolved components in the prepolymer solution (Figure 2A). With higher PVA-NB:SS-DT content, a decrease in *G′* before the irradiation step was observed, leading to increased mechanical support for IPN5 before the photopolymerization. Additionally, we observed that the reduced irradiation times (1–5 min) and variation of the light source and light intensity did not influence the gel formation (Figures S7, S8, Tables S5, and S6).

**Figure 2:**
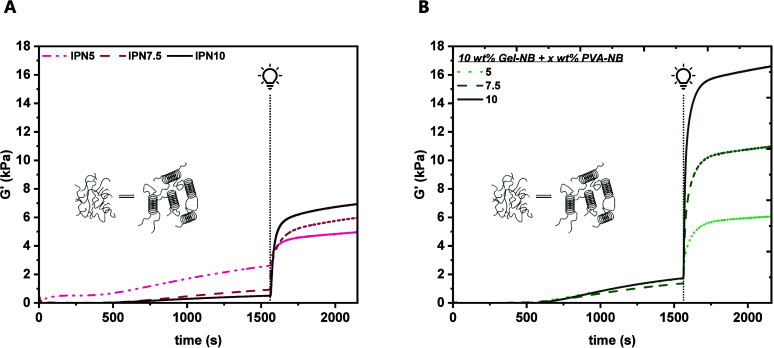
Representative storage moduli (*G*′) over time for (A) IPNs containing 10 wt % Gel +5–10 wt % PVA-NB:SS-DT and (B) hybrid materials containing 10 wt % Gel-NB:SS-DT + 5–10 wt % PVA-NB:SS-DT. The physical gelation stage and the irradiation are indicated. 0.6 mM Li-TPO was used as the PI.

The prepared semi*-*IPN hydrogels are hydrophilic polymers with various degrees of cross-linking. Due to the interactions between a hydrophilic network and water molecules, the materials can retain water and also swell due to further water uptake.^39^ Although water uptake is a necessary feature for such materials, uncontrollable absorption of water complicates the preparation of form-stable hydrogels.^5^ During photorheological measurements, it was shown that adding gelatin to the pristine PVA-NB enhances the gel stiffness, indicating a higher overall cross-linking density. Thus, different swelling behavior compared to independent PVA-NB:SS-DT and gelatin networks is expected. For all swelling experiments, samples produced during the photorheology study (disks, Ø 8 mm, thickness: 0.5 mm) were immersed in PBS for 24 h and weighed (swollen weight), and the dry weight was obtained after lyophilization of the samples. A direct correlation between cross-linking density and swelling ratio was determined (Figure 3A). Generally, samples with a higher macromer concentration showed lower water absorbency than those with a lower degree of cross-linking. Interestingly, semi*-*IPNs exhibited a lower MSR (MSR or q22) (IPN5: 20.4 ± 1.9, IPN7.5: 17.1 ± 2.1, IPN10: 14.3 ± 2.9) related to the independent networks. Compared to the pure TEC network, a decrease of around 50% for all PVA-NB:SS-DT concentrations (5 wt %: 33.8 ± 2.0, 7.5 wt %: 26.5 ± 0.9, 10 wt %: 16.9 ± 1.5) was observed. Interestingly, the IPNs showed substantially lower MSR than neat gelatin hydrogels (10 wt % in PBS, MSR: 26.6 ± 3.8), regardless of the final macromer concentration and cross-linking density. The combination of intramolecular triple helix formation (Gel) and additional intermolecular hydrogen bonds (PVA and Gel) is anticipated to add superior network connectivity, especially in the case of low PVA scaffold concentrations.

**Figure 3:**
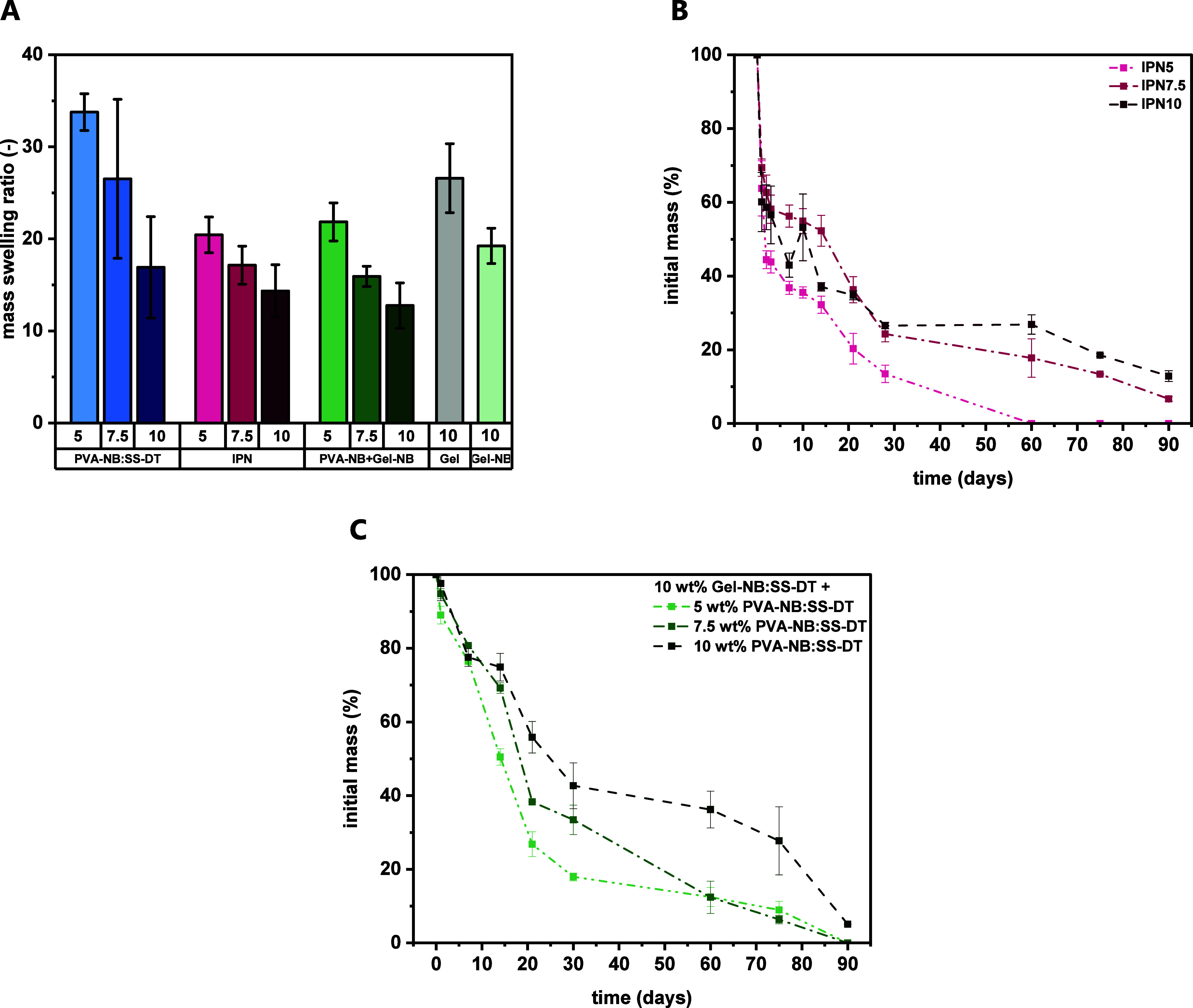
(A) Mass swelling ratio (MSR, 24 h in PBS, 21 °C) of hydrogels derived from 5 to 10 wt % PVA-NB:SS-DT (blue) and upon the addition of 10 wt % gelatin (semi-IPNs, pink) or 10 wt % Gel-NB (green). Reference materials (10 wt % Gel, 10 wt % Gel-NB:SS-DT) are depicted. (B) In vitro degradation of IPN5, IPN7.5, and IPN10 under physiological conditions (37 °C, pH 7.4). (C) In vitro degradation of hybrid gels containing 10 wt % Gel-NB:SS-DT and 5–10 wt % PVA-NB:SS-DT under physiological conditions (37 °C, pH 7.4).

After the material’s physicochemical properties were assessed, the long-term stability of semi*-*IPNs was analyzed via an in vitro degradation study mimicking physiological conditions (37 °C, pH 7.4). Within the hydrogels, two principal hydrolytic degradation routes (Figure S9) seem plausible. On the one hand, PVA-NB and the cross-linker SS-DT contain ester bonds that are hydrolytically labile.^40^ Furthermore, gelatin is a collagen-derived protein, and its peptide bonds are prone to hydrolysis, especially under elevated temperatures.^41^ Interestingly, around 40% mass loss was observed for all samples within the first 24 h (Figure 3B and Table S7). This corresponds approximately to the gelatin content of the semi-IPNs. Thus, gelatin leached out of the materials within the first day, as it lacks freely accessible thiol groups on its primary structure for thiol–norbornene coupling.^30^ Thereafter, mainly the PVA-NB:SS-DT network was present, and in vitro degradability within the examined time frame was shown. As a reference, neat in vitro degradability of PVA-NB:SS-DT was assessed (Figure S5 and Table S3), and comparable hydrolytic degradation was shown. All samples showed distinctive swelling, and as the initial disc shape was not retained (Figures S11–S13), bulk degradation was postulated for all samples.^42^ IPN5, which has the lowest cross-linking density of all tested materials, degraded completely within 60 days. By increasing the network density, decelerated degradability was observed. Residual masses of 7 and 12% were found for IPN7.5 and IPN10, respectively. Generally, the cross-linking density was reduced over time, leading to a softening of all networks. This phenomenon was accompanied by higher swellability of the material (Figure S10 and Table S7) and volumetric expansion. Thereby, water molecules can more easily penetrate into the material, leading to faster degradation^40^ for IPN5 compared to the more densely cross-linked IPN7.5–10. However, compared to the neat PVA-NB:SS-DT, lower swellability and thus superior stability have been achieved (Figure S5 and Table S3) for all IPNs.

### Network Stabilization by Hybrid PVA- and Gel-NB Hydrogels

The incorporation of disulfide-based linkers into PVA-NB networks delivered modular material platforms upon the addition of 10 wt % gelatin. Both mechanical stability and form stability were enhanced by the presence of physically cross-linkable gelatin matrices within pure synthetic PVA-NB:SS-DT materials. However, in vitro degradation experiments of semi-IPNs showed that the gelatin matrices tend to leach out of the material at 37 °C, and thus, covalent insertion of the gelatin matrix seemed feasible. In the literature, norbornene-modified gelatin (Gel-NB) exhibited excellent physical gelation properties, as well as the ability for subsequent thiol–ene photopolymerization. Therefore, we decided to substitute unmodified gelatin with the same amount of Gel-NB (DS = 0.217 mmol g^–1^). Thus, creating hybrid PVA-NB/Gel-NB:SS-DT gels should still show strong hydrogen bonds between the macromer chains. Before incorporation of Gel-NB into hybrid gels containing PVA-NB:SS-DT, the mechanical and photopolymerization behavior of Gel-NB:SS-DT was investigated using in situ photorheology (Figure S14 and Table S8). Excellent photoreactivity (*t*_D_: 1.23 s) and gelation behavior (*G′*_max_ *:* 1.74 kPa) were determined, without sacrificing the ability for physical gelation prior to TEC.

Next, prepolymer formulations containing 5–10 wt % PVA-NB, 10 wt % Gel-NB, and an equimolar amount (with respect to norbornene functional groups) of the dithiol SS-DT were analyzed via in situ photorheology (Figure 2B and Table S9) using the same measurement procedure previously presented for *semi-*IPNs. Similar to the trends observed for semi-IPNs, higher gel stiffness (5 wt %: 6.0 kPa, 7.5 wt %: 10.5 kPa, 10 wt %: 16.4 kPa) and shorter delay times (5 wt %: 1.5 s, 7.5 wt %: 1.8 s, 10 wt %: 0.3 s) were observed with higher PVA-NB:SS-DT content. Interestingly, regardless of the concentration of the PVA-based components, similar gel stiffness (1.5 kPa) after the gelatin physical cross-linking was obtained. Thus, it was shown that the addition of Gel-NB had a positive effect on the material properties and photoreactivity compared to those of semi-IPNs.

Thereafter, we investigated the mass swelling of the produced hybrid hydrogels and compared the MSR to that of semi*-*IPNs (Figure 3A). As previously discussed, by the incorporation of 10 wt % unmodified gelatin (IPNs), lower mass swelling ratios compared to the independent networks (PVA-NB:SS-DT, Gel) were observed. However, exchanging Gel for Gel-NB exhibited MSRs of 21.8 (5 wt % PVA-NB, 10 wt % Gel-NB), 15.9 (7.5 wt % PVA-NB, 10 wt % Gel-NB), and 12.8 (10 wt % PVA-NB, 10 wt % Gel-NB). Semi-IPNs with the same amount of PVA-NB and unmodified gelatin display similar MSR values between 20.4 (IPN5) and 14.3 (IPN10). Considering that the overall cross-linking density (higher *G′*_max_) was increased by the chemical incorporation of Gel-NB, the mass swelling ratio was merely marginally reduced compared to IPNs. Nevertheless, by the modification of gelatin with carbic anhydride, the norbornene moiety is attached together with highly hydrophilic and polar carboxylic groups. Thus, the overall network became more hydrophilic, and higher water affinity can be expected. As the MSR was quite similar to the IPNs, the more hydrophilic functionalities counterbalanced the higher cross-linking density and led to no considerable variation of the swelling properties.

Next, we aimed to study the hydrolytic degradation of hybrid gels. Compared to *semi*-IPNs, the gelatin backbone was covalently bound via the cross-linker SS-DT. Thereby, leaching of the gel matrix should be suppressed. Similar to the first study, degradation was monitored over a period of 90 days, and the degradation was monitored over mass change (Figure 3C and Table S10) and swelling properties (Figure S15). As expected from previous degradation experiments, the cross-linking density had a direct influence on the hydrolytic degradability. Within the targeted 90 days of the study, all tested macromer concentrations showed gradual in vitro degradation, resulting in either complete network hydrolysis (5–7.5 wt % PVA-NB) or a residual of 5% of initial dry mass (10 wt % PVA-NB). Noteworthy, for all specimens, no premature gelatin leaching was observed, as the dry mass after 24 h remained fairly constant (2–5%) for all prepared hydrogels. Thus, the chemical incorporation of Gel-NB into the PVA-NB:SS-DT networks led to stabilized networks that suppressed premature material loss.

### Two-Photon Micropatterning of Disulfide-Bearing Hydrogels

Disulfide-containing hydrogels have been degraded either in the one-^11^ or two-photon regime^5^ in recent years. Herein, the neat PVA-NB:SS-DT hydrogels were degraded using high concentrations (>5 mM) of the PI Li-TPO via radical-mediated disulfide fragmentation during in situ photorheology. We furthermore demonstrated that hydrogel formation was favored over degradation at low Li-TPO concentrations (<1 mM). Next, we investigated the 3D micropatterning of the presented *semi-*IPN and hybrid hydrogels by means of two-photon (2P) irradiation. Therefore, we added the water-soluble cyclic benzylidene ketone-based two-photon sensitizer P2CK (0.5–2.0 mM, Figure 4)^43^ to the photopolymerized hydrogels. Thereby, the absorbance in the 2P regime should be promoted to facilitate disulfide cleavage. Micropatterning experiments were conducted using 5 wt % PVA-NB:SS-DT hydrogels containing either 10 wt % Gel (IPN5) or 10 wt % Gel-NB:SS-DT. After fabrication, the patterned channels were washed with PBS and swollen with a high-molecular-weight fluorescent dextran (2000 kDa, FITC2000). However, FITC2000 infiltrates only the open microchannels (Figure 4). Thereby, visualization of the eroded channels in the bulk hydrogels is facilitated, as the high molecular weight of FITC2000 prevents diffusion of FITC2000 into the bulk hydrogel.

**Figure 4:**
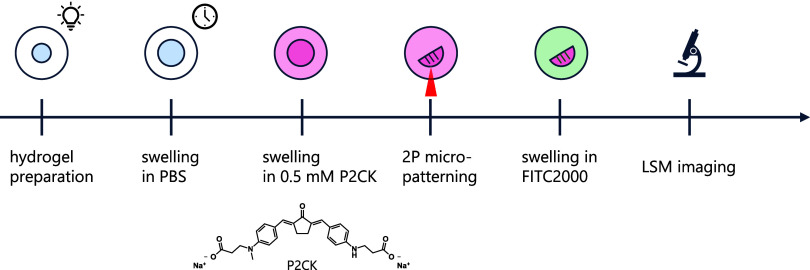
Schematic overview of hydrogel formation and 2P micropatterning. Prior to the 2P degradation, hydrogels were formed via UV-induced thiol–ene polymerization and swollen for 18 h until reaching equilibrium swelling. Thereafter, the 2P sensitizer P2CK was added, and hydrogels were micropatterned using a fs-pulsed laser. Microchannel formation was visualized by infiltration after swelling in fluorescent dextran via confocal microscopy.

In the first preliminary studies, 2P micropatterning was performed with IPN5, wherein the chromophore (P2CK) concentration was varied (0.5–2.0 mM, Figures S17–S19). Although all tested P2CK concentrations displayed minimal variations from the theoretical channel widths (at all tested scanning speeds and laser powers), gels containing 1.0–2.0 mM P2CK exhibited high standard variations in the mean fluorescence intensity, indicating an inhomogeneous fluorescence signal distribution along the channel length. Therefore, we selected a concentration of 0.5 mM P2CK for further tests. For 2P micropatterning of the IPN5 hydrogel (Figure 5A), open channels could be formed with laser intensities as low as 20 mW (scanning speed: 400 mm s^–1^). However, at the faster scanning speeds (500–600 mm s^–1^), a higher laser intensity threshold for network degradation was observed (30 mW). Without the addition of P2CK, no fluorescence was observed in the scanned regions (Figure S16). Hence, we demonstrate that 2P cleavage can happen only in the presence of a 2P-active chromophore. Next, to assess the channel quality, we divided the fabricated channels into 3 equal segments and measured channel widths (Figure 5C). Regardless of the scanning speed and laser power, minimal variations (less than 15%) were observed in the channel width of IPN5. Low standard deviations furthermore indicate a homogeneous channel formation.

**Figure 5:**
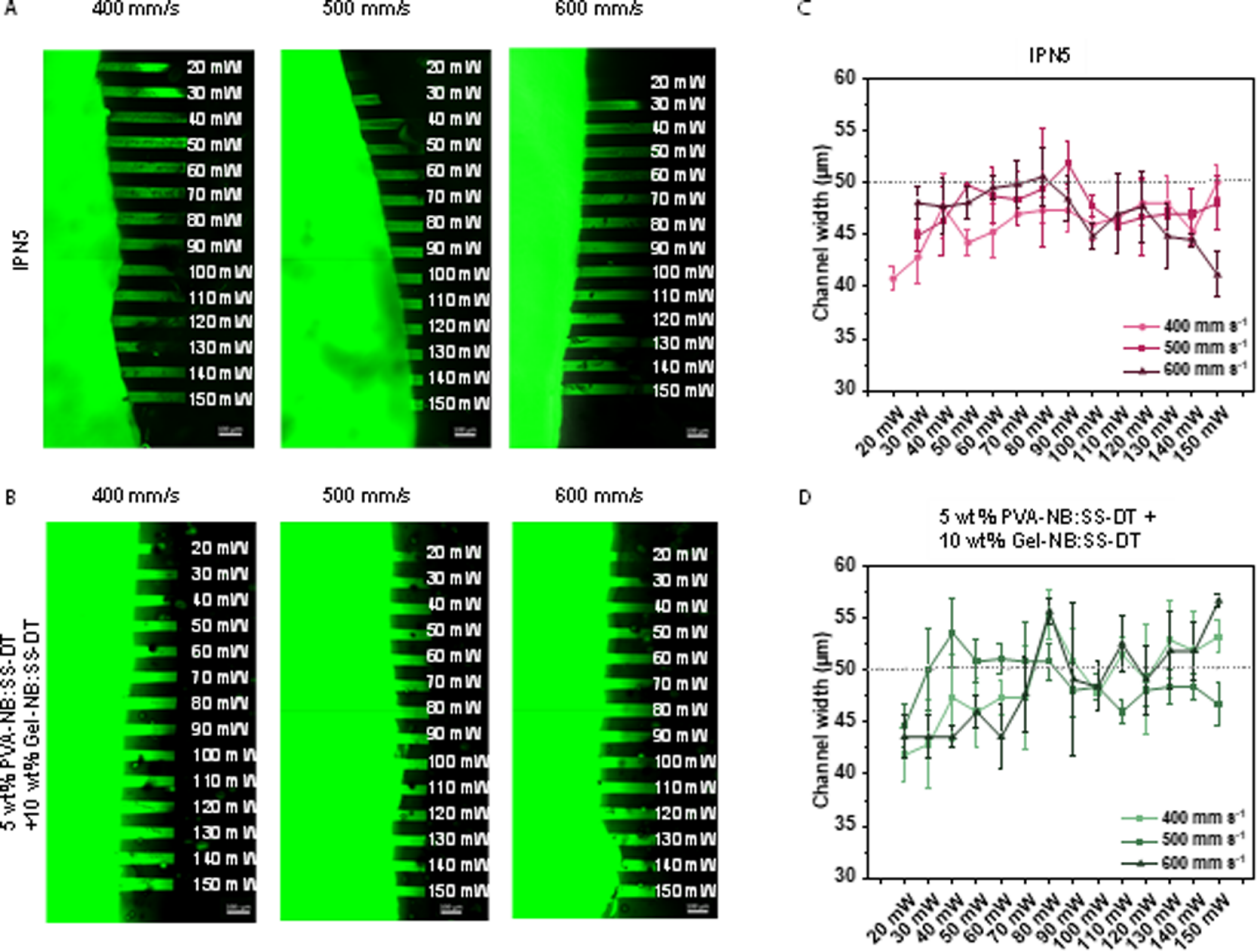
Microchannels were fabricated by two-photon degradation of disulfide-based (A) IPN5 and (B) disulfide-containing hybrid hydrogels (5 wt % PVA-NB:SS-DT+10 wt % Gel-NB:SS-DT) in the presence of two-photon initiator P2CK (0.5 mM). Individual *x*,*y*-planes were scanned with either variable laser power of 20–150 mW or scanning speeds of 400–600 mm s^–1^. Thereafter, hydrogels were soaked in a solution of high-molecular-weight fluorescent dextran (FITC2000) for 18 h, and microchannels were visualized by confocal microscopy. Scale bar: 100 μm. Channel widths of (C) IPN5 and (D) hybrid gels compared to the theoretical channel width (50 μm, dotted line).

However, comparing the fluorescence intensity of the channels in IPN5 with the surroundings (PBS solution, bright green), only partial network degradation can be observed, as the penetration of a high-molecular-weight fluorescent dextran was chosen as a parameter for the success of the micropatterning. For IPN5, the normalized fluorescence intensity (Figure 6) was below 50% (with the exception of 30 mW at 500 mm s^–1^). Even prolonged treatment (48 h) with FITC2000 did not lead to better infiltration, which could result from unsuccessful removal of the eroded material. In the tested hydrogels, gelatin was only physically incorporated into the *semi-*IPN. However, at *T* > 37 °C, gelatin starts to soften and disentangle from the network. Irradiating the hydrogels with a fs-pulsed laser, even at low intensities, will eventually cause local heat production, thus leading to the melting and dissolving of the gelatin backbone. Thus, after cooling back to room temperature, physical gelation of gelatin leads to partial clogging of the channel, as displayed by a lower fluorescence signal compared to the gel’s surroundings.

**Figure 6:**
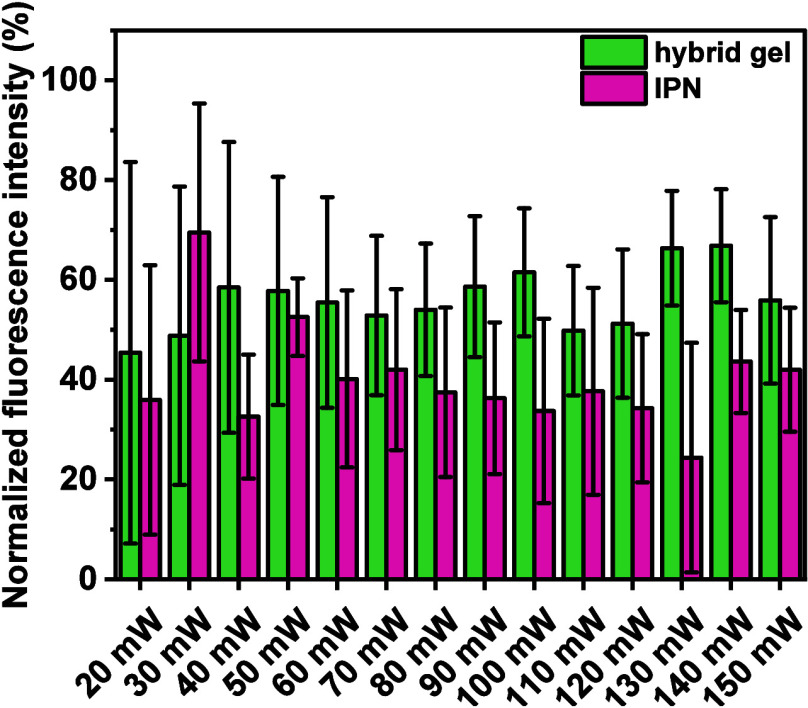
Normalized fluorescence intensity of IPN5 (pink) and the hybrid material (green, 5 wt % PVA-NB:SS-DT + 10 wt % Gel-NB:SS-DT). The fluorescence signal of FITC2000 within the channels was normalized to the surrounding (PBS, no hydrogel). The channels were divided into three equal segments, and thereof, the average fluorescence intensity and standard deviations were calculated.

To counteract the clogging of the formed microchannels, we introduced Gel-NB:SS-DT instead of the unmodified Gel. Thereby, the norbornene-modified gelatin should remain stable during laser exposure, as Gel-NB is chemically incorporated via thiol–ene cross-linking. Two-photon micropatterning experiments were performed similar to IPN5 (scanning speed: 400–600 mm s^–1^, laser power: 30–150 mW). Similar to previous observations made for IPN5, no microchannels were formed for unsensitized (no P2CK) materials. Immersion of the hydrogels in 0.5 mM P2CK prior to the 2P micropatterning led to the successful formation of microchannels, as the formed channels could be visualized with the fluorescent dye (Figure 5B). Herein, perfusable channels were produced at laser powers of as low as 20 mW (400–500 mm s^–1^). For the highest scanning speed of 600 mm s^–1^, only partially degraded channels were obtained. However, for all tested laser intensities, the full length of the eroded channel could be perfused by FITC2000, demonstrating successful cleavage of the disulfide cross-linkers within the hydrogels. Additionally, the widths of all fabricated channels (Figure 5D) showed little variations (<10%) from the theoretical value of 50 μm. No clogging of the channels due to gelatin gelation was observed, as displayed by higher normalized fluorescence intensities (Figure 6). As a higher fluorescence signal correlates with less cross-linking density, enhanced 2P degradation was obtained for the hybrid gels compared to IPN5. With higher laser intensities, the microchannels appear to have sharp and distinct boundaries. Nevertheless, small amounts of the dye can be found in close proximity to the formed channels. It is assumed that upon disulfide cleavage, the system becomes dynamic. Thereby, disulfide bonds in close vicinity to the microchannels could interact with formed thiyl radicals,^5^ allowing for the penetration of FITC2000 into the bulk hydrogels. Finally, the micropatterned gels (IPNs and hybrid gels) were analyzed 7 days after fabrication to gain insights into their respective long-term stability (Figure S21). As expected, an increase in the channel width was observed for both materials (Figure S22), resulting from swelling of the hydrogels. However, the channel’s geometry was well preserved, and no significant clogging was identified. Interestingly, the normalized fluorescence intensity increased for IPN5 (Figure S23), whereas the normalized fluorescence slightly decreased for the hybrid gel. The normalized fluorescence was calculated from the material’s fluorescence surrounding the channels. For the hybrid channels, we observed higher background fluorescence stemming from diffusion of the dye into the dynamic network compared to day 1 (Figure S21). This contributes to an apparent decrease in normalized fluorescence. However, the hydrogels analyzed herein show excellent long-term stability as the fabricated microchannels retain their original shape without any visible blocking of the structures.

## Conclusions

With this study, we aimed to develop novel strategies for two-photon degradation of disulfide-based hydrogels with increased network stability. Therefore, we developed a simple disulfide-bearing cross-linker SS-DT from readily available starting materials. We analyzed the network formation between PVA-NB and SS-DT via light-induced thiol–ene cross-linking and showed that already a low radical PI concentration (0.6 mM) leads to network formation. However, at high amounts of photogenerated radicals (17 mM), disulfide cleavage counteracts the network build-up. Upon the addition of unmodified gelatin, *semi*-IPNs were fabricated that showed both variable gel stiffness (5–7 kPa) and improved network stability during swelling experiments. However, in vitro hydrolytic degradation experiments showed poor long-term stability of the materials as the unmodified gelatin backbone leached out of the hydrogels within the first 24 h. Therefore, gelatin was norbornene-modified to yield Gel-NB. Subsequent hybrid photo-cross-linking with PVA-NB and SS-DT broadened the material properties even further (*G*′_final_ ∼ 5–17 kPa). Interestingly, the mass swelling ratios of the independent networks could be reduced by up to 50% via strong intermolecular hydrogen bonds, giving hydrogels with higher volumetric form stability. Additionally, tunable hydrolytic in vitro degradation (∼90 days) without premature material loss was observed. Finally, two-photon degradation of both *semi*-IPNs and PVA-NB:SS-DT/Gel-NB:SS-DT hybrid gels was demonstrated by means of microchannel fabrication. We demonstrated the importance of the chemical incorporation of both PVA and gelatin macromers on microchannel perfusion. This study extends the scope of easily accessible and cost-efficient photolabile cross-linkers for a variety of material classes in a controllable, mild way by the use of UV light. The dynamic nature of the disulfide bond allows for engineering of tunable materials with tailored properties for applications such as drug delivery or microfluidics.

## Supplementary Material



## Notes

^1^H NMR spectra of all synthesized macromers and cross-linkers, as well as the ^13^C NMR spectrum of SS-DT; calculation of the DS for PVA-NB and Gel-NB; detailed information on the in situ photorheological measuring sequence; optimization of the irradiation time and light source for in situ photorheological measurements of IPN5 and in situ photorheology measurements of Gel-NB:SS-DT (10 wt %); additional MSRs for hydrolytic degradation studies and proposed hydrolytic degradation of PVA- and gelatin-derived networks; additional confocal microscopy imaging of micropatterned hydrogels; and raw data of all presented graphs available at TU Wien Research Data (PDF)
