# Supplementary material for: Highlighting the Influence between Physical and Chemical Cross-Linking in Dynamic Hydrogels for Two-Photon Micropatterning
Source: Biomacromolecules. 2025 May 9;26(7):4084–94. doi: 10.1021/acs.biomac.5c00062 (PMC12264952; doi:10.1021/acs.biomac.5c00062)
Supplement: Supplementary file 1 [file bm5c00062_si_001.pdf]

Supporting Information:

Highlighting the influence between physical and chemical crosslinking in dynamic hydrogels for two-photon micropatterning

Antonella Fantoni[1,2], Alice Salvadori[2,3], Aleksandr Ovsianikov[2,3], Robert Liska[1] and Stefan Baudis[1,2,4]*

[1]Institute of Applied Synthetic Chemistry, Technische Universität Wien, Getreidemarkt 9, 1060 Vienna, Austria

[2]Austrian Cluster for Tissue Regeneration, 1200 Vienna, Austria

[3]Institute of Materials Science and Technology, Technische Universität Wien, Getreidemarkt 9, 1060 Vienna, Austria

[4]Christian Doppler Laboratory for Advanced Polymers for Biomaterials and 3D Printing, Getreidemarkt 9, 1060 Vienna, Austria

*Correspondence: Stefan.baudis@tuwien.ac.at

1. NMR SPECTRA AND CALCULATION OF DS OF SYNTHESIZED COMPOUNDS

1.1. PVA-NB:

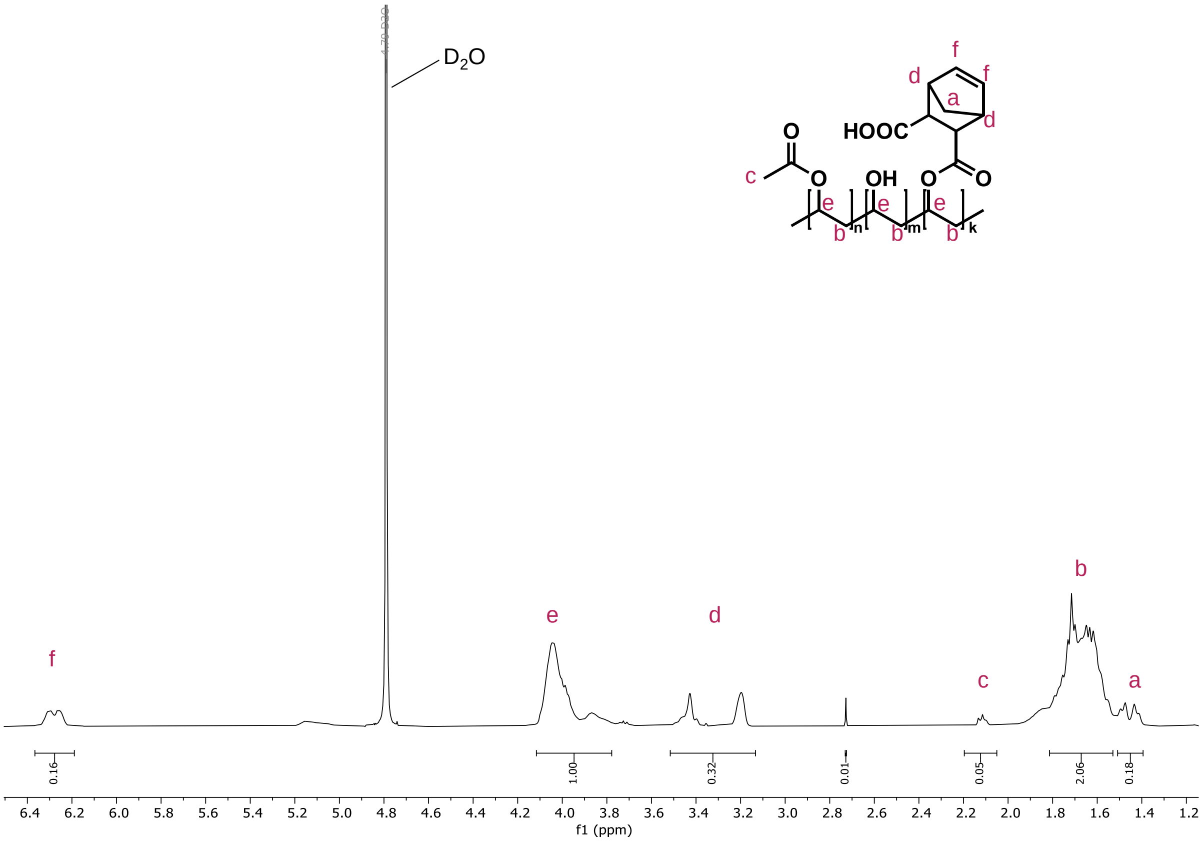

Figure S 1: ^{1}H-NMR spectrum of PVA-NB in D_2O. Corresponding peaks are allocated.

The degree of substitution was calculated with the following equation:

$$DS\ (\%) = \frac{\frac{Integral(NB\ double\ bond, \sim 6{,}3\ ppm)}{2}}{Integral(PVA - CH,\ \sim 4{,}0\ ppm)} * 100\%$$

1.2. Gel-NB:

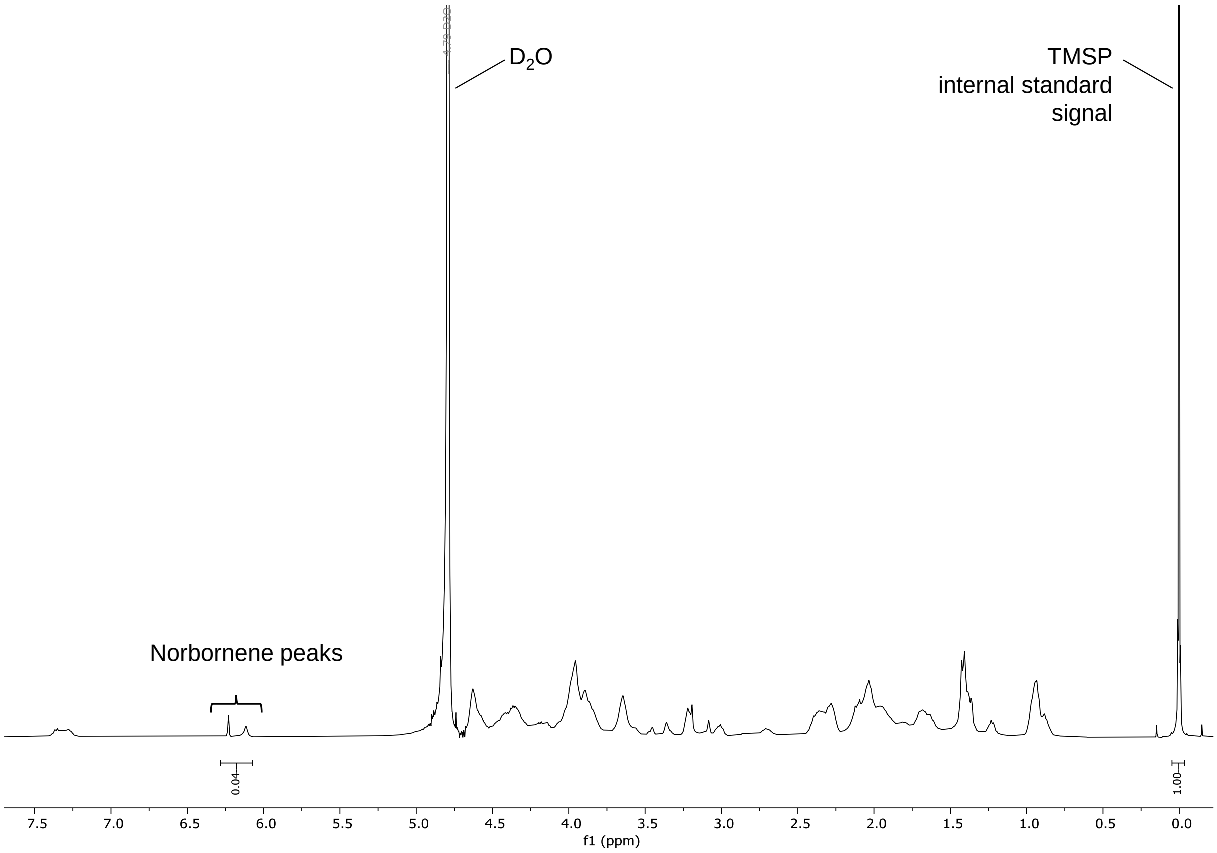

Figure S 2: ^{1}H-NMR spectrum of Gel-NB in D_2O. Corresponding peaks are allocated.

For the determination of the DS, TMSP was dissolved in D_2O (1 mg mL^{-1}, 0,00348 mmol). Thereafter, Gel-NB (2.88 mg) was dissolved in 600 µL of the TMSP stock solution. The DS was calculated with the following equation:

$$DS(mmol\ g^{-1}) = \frac{\frac{Integral(NB\ double\ bonds,\ \sim 6{,}22\ ppm) * n_{TMSP}(mmol)}{2}}{\frac{Integral(TMSP\ methyl\ protons, 0\ ppm) * m_{Gel-NB}(g)}{9}}$$

1.3. SS-DT:

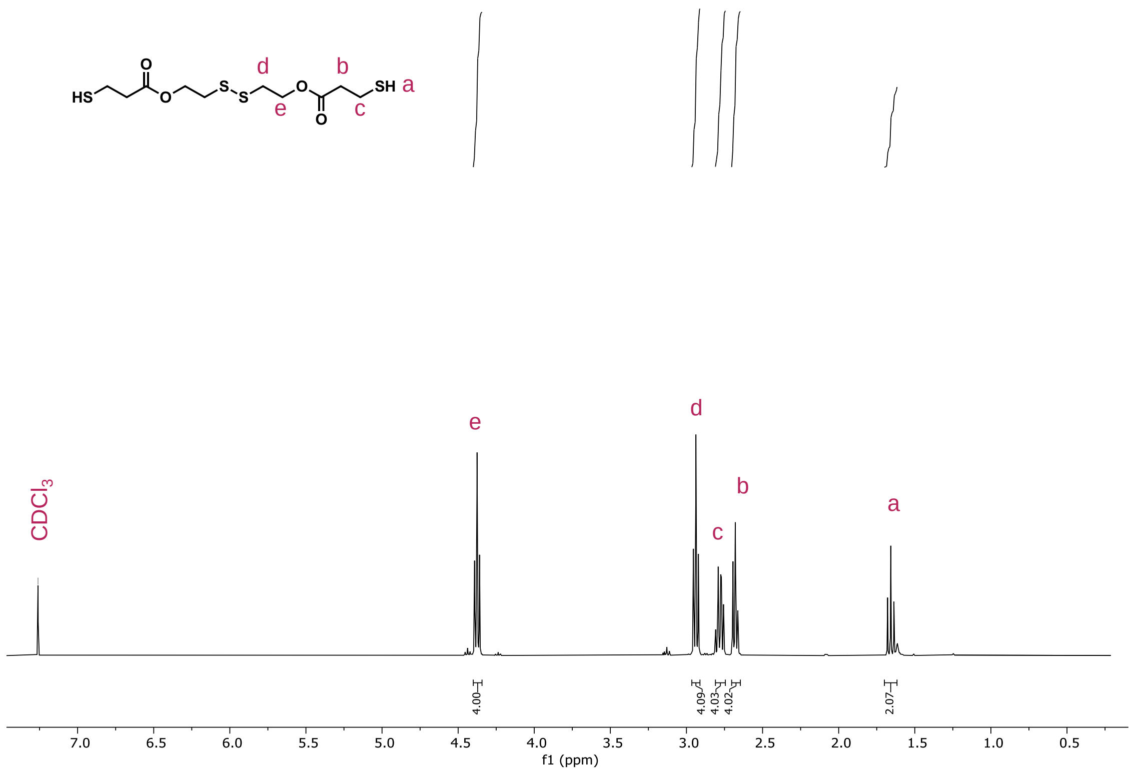

Figure S 3: ^{1}H-NMR spectrum of SS-DT in $CDCl_3$. Corresponding peaks are allocated.

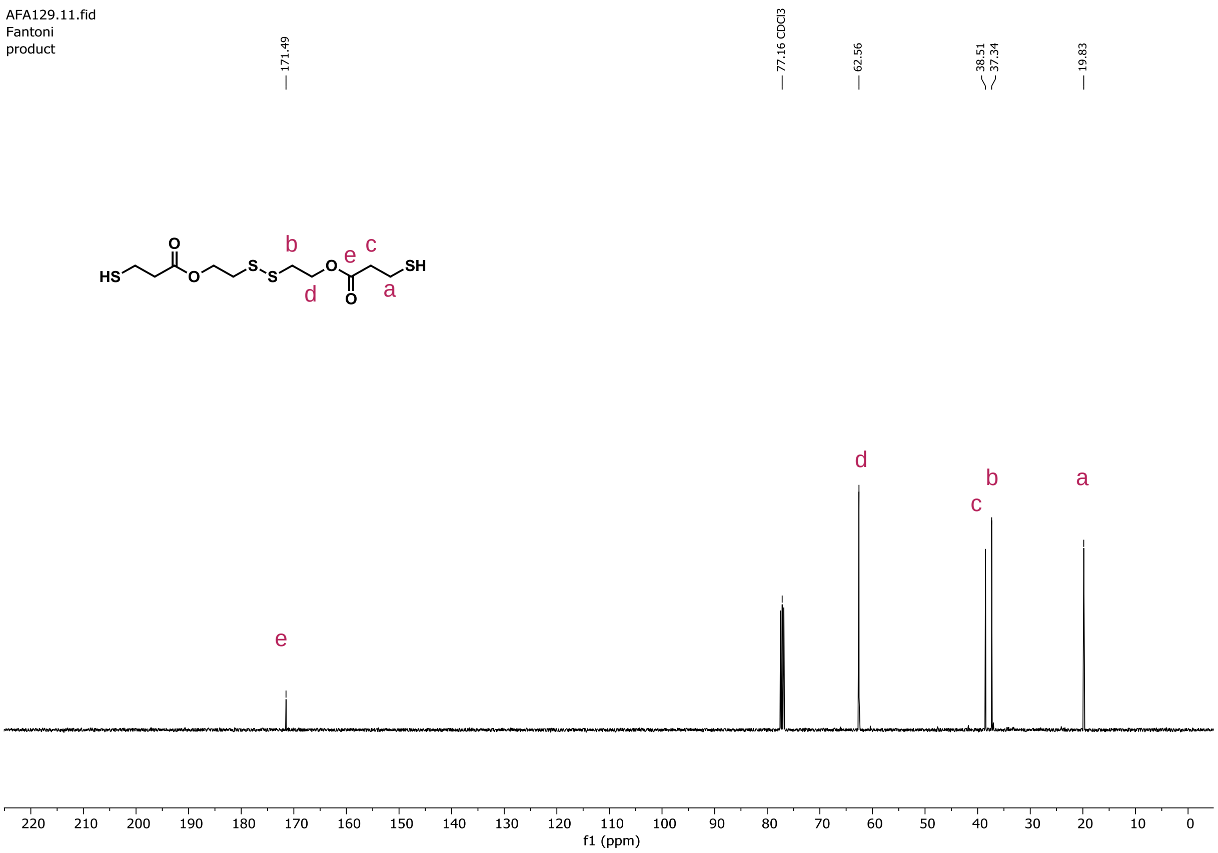

Figure S 4: ^{13}C-NMR of SS-DT in $CDCl_3$.Corresponding peaks are allocated.

2. OPTIMIZING THE THIOL-ENE PHOTOPOLYMERIZATION

2.1. *In-situ* photorheology of PVA-NB:SS-DT

Table S 1: Results of the photorheological analysis of 5-10 wt% PVA-NB: SS-DT in PI-PBS, Li-TPO (17 mM in PBS) was used as PI.

	G'_{max} (kPa)	t_D (s)	ΔG'/Δt (s)
5 wt%	1.51 ± 0.07	5.47 ± 0.61	14.5 ± 0.1
7.5 wt%	4.05 ± 0.42	0.55 ± 0.37	114 ± 30
10 wt%	9.78 ± 1.77	0.44 ± 0.15	771 ± 232

Table S 2: Results of the photorheological analysis of 10 wt% PVA-NB:SS-DT in PI-PBS. Li-TPO (0.2-17 mM in PBS) was used PI.

Li-TPO (mM)	t_D (s)	ΔG'/Δt (Pa/s)	G'_{max} (kPa)
0.2	18.2 ± 6.2	9.44 ± 2.18	0.45 ± 0.12
0.4	10.1 ± 1.9	19.3 ± 5.5	0.55 ± 0.21
0.6	3.26 ± 0.07	311 ± 2	4.43 ± 1.89
1	2.66 ± 0.09	268 ± 3	3.38 ± 0.48
5	0.66 ± 0.01	292 ± 28	4.71 ± 0.34
17	0.44 ± 0.15	772 ± 23	9.78 ± 1.77

2.2. *In-vitro* degradation of PVA-NB:SS-DT

*Table S 3: In-vitro degradability of semi-IPNs composed of 5-10 wt% PVA-NB:SS-DT and 10 wt% gelatin: results of the degradation study at 37 °C. *) Samples too fragile to be weighed. **) samples fully degraded in the observed timeperiod.*

	IPN5		IPN7.5		IPN10	
time (days)	MSR (-)	initial mass (%)	MSR (-)	initial mass (%)	MSR (-)	initial mass (%)
1	52.8 ± 1.8	99.7 ± 2.7	45.0 ± 0.3	100 ± 0.7	40.3 ± 5.3	98.7 ± 1.1
2	66.0 ± 0.4	68.7 ± 3.0	50.1 ± 6.1	78.2 ± 4.5	41.7 ± 4.5	83.6 ± 1.4
3	85.8 ± 0.6	68.0 ± 0.1	48.9 ± 3.7	73.7 ± 3.7	41.6 ± 3.6	81.5 ± 2.1
7	121 ± 1	61.0 ± 1.6	81.7 ± 1.4	71.9 ± 3.6	70.7 ± 3.5	67.9 ± 3.4
10	*	59.8 ± 2.7	83.4 ± 1.0	70.5 ± 6.3	73.6 ± 6.2	78.1 ± 0.9
14	*	56.4 ± 7.4	95.6 ± 13.3	67.9 ± 2.5	88.0 ± 2.5	62.0 ± 2.9
21	*	44.2 ± 0.7	98.2 ± 7.0	51.9 ± 1.4	95.1 ± 2.1	59.7 ± 0.2
28	*	27.5 ± 0.6	110 ± 6	39.9 ± 2.2	105 ± 2	51.4 ± 7.8
60	*	1.57 ± 7.8	*	33.4 ± 7.0	*	51.8 ± 4.1
75	**	**	*	22.3 ± 4.7	*	43.4 ± 3.6
90	**	**	*	14.6 ± 2.0	*	27.8 ± 1.1

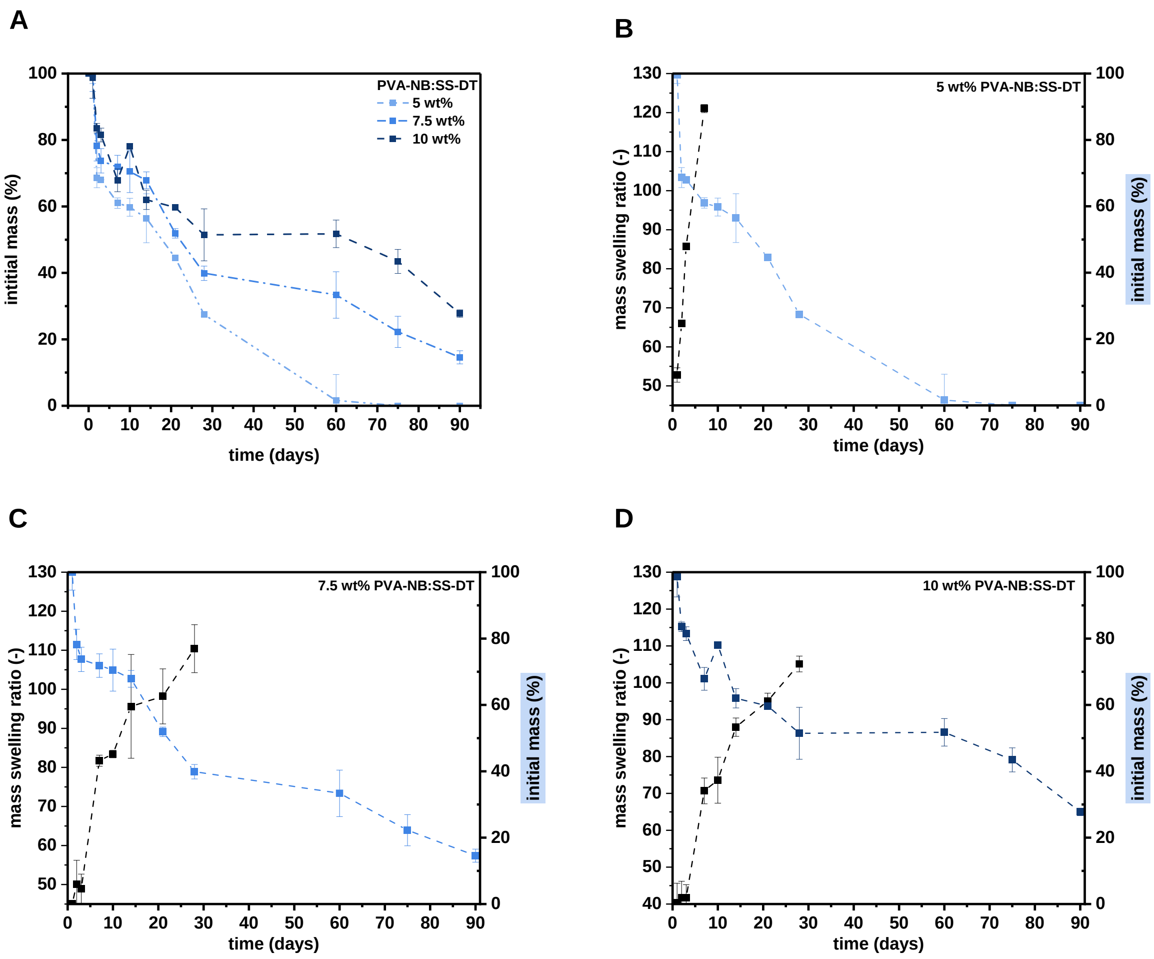

Figure S 5: A) Overview of the in-vitro degradability of hydrogels at 37 °C containing B) 5 wt%, C) 7.5 wt% and D) 10 wt% PVA-NB:SS-DT. Mass change of dry mass (initial mass, %) and mass swelling ratio (MSR, -) are plotted over time. MSR was recorded until the samples became too fragile to examine.

3. PREPARATION AND CHARACTERIZATION OF *SEMI*-IPNs

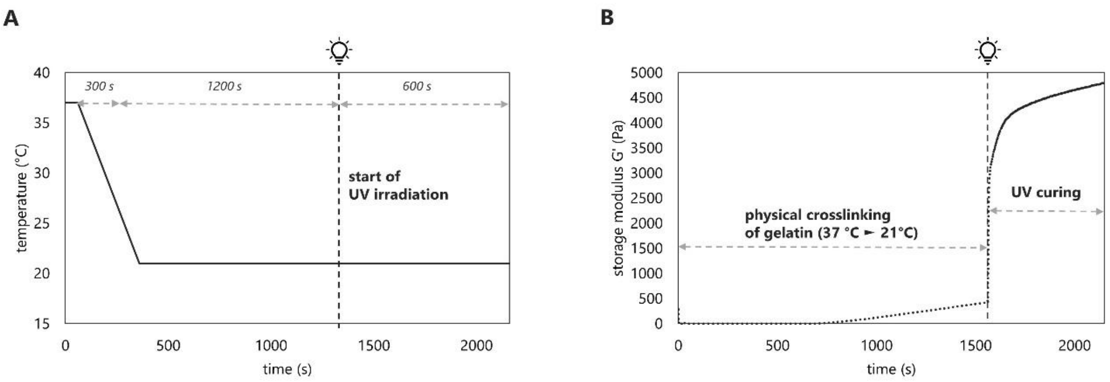

Figure S 6: Crosslinking sequence for the preparation of IPNs. (A) Temperature profile for in-situ photorheological measurements for physical crosslinking to UV crosslinking with cooling from 37 °C to 21 °C (5 min), holding at 21 °C for 20 min followed by UV crosslinking for 10 min at 21 °C. (B) Exemplary development of the storage modulus over time for semi-IPN crosslinking. Markers indicate the physical and UV crosslinking sections.

Table S 4: Results of the photorheological analysis of semi-IPNs prepared containing 5-10 wt% PVA-NB:SS-DT in PI-PBS and 10 wt% Gel in PBS. 0.6 mM Li-TPO in PBS were added as photoinitiator.

	t_D (s)	ΔG'/Δt (kPa s^{-1})	G'_{max} (kPa)
IPN5	3.58 ± 0.27	85.7 ± 2.7	4.99 ± 0.14
IPN7.5	3.40 ± 0.15	393.4 ± 4.76	5.81 ± 0.26
IPN10	2.90 ± 0.05	554.8 ± 8.4	6.98 ± 0.04

3.1. Optimizing the photopolymerization: variation of the irradiation time

Photorheological analysis of one representative IPN system was conducted to investigate the influence of variable irradiation time on the final hydrogel properties. IPN5 was chosen as the model system for this study. In contrast to the "standard" measurements (10 min UV exposure), the irradiation time was lowered to 1 min and 5 min, respectively (Figure S 7, Table S5). However, the overall measurement time for the UV-crosslinking step was kept constant, so that after 1 min of irradiation a 9 min "dark" reaction was recorded. For the sample with 5 min of irradiation time, 5 min of "dark" reaction were monitored.

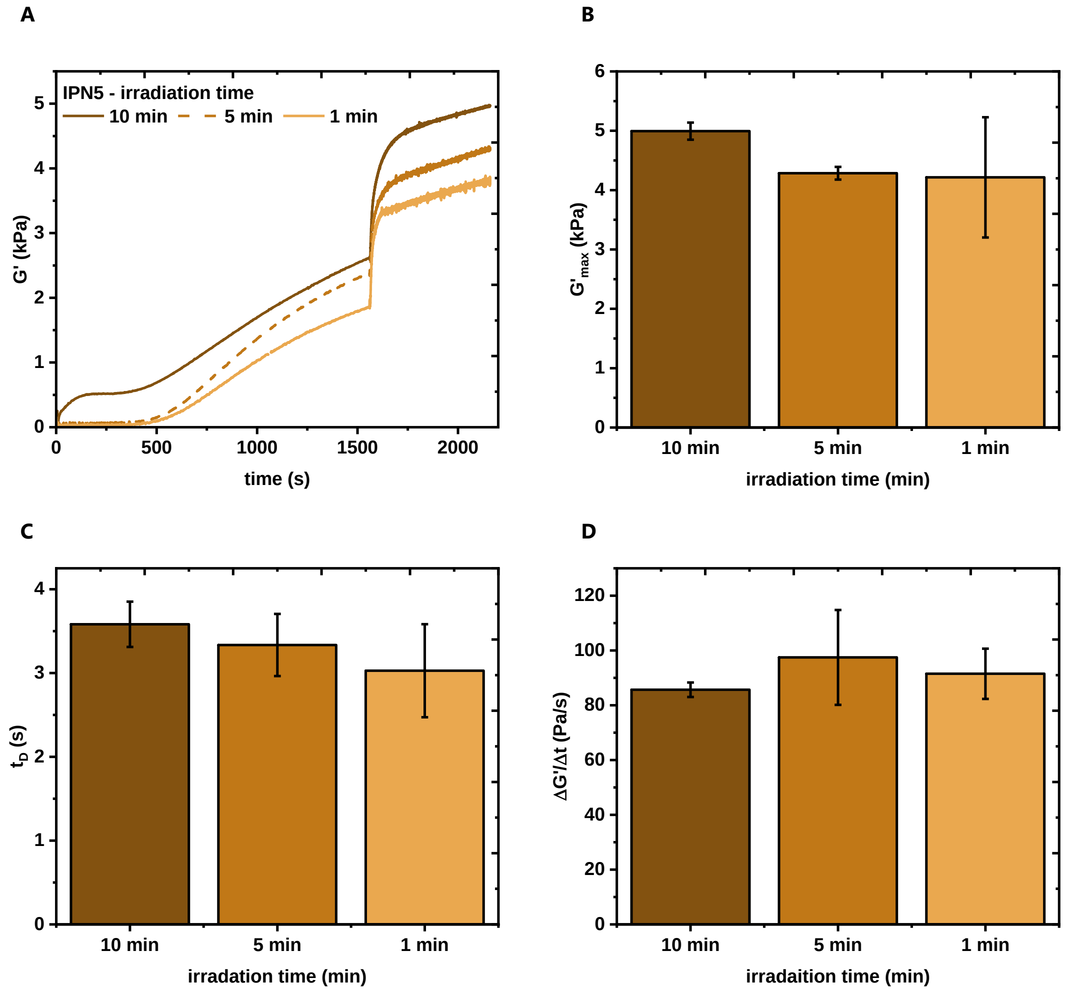

Figure S 7: Results of the photorheological analysis for IPNs composed of 10 wt% gelatin and 5wt% of PVA-NB:SS-DT (IPN5). Photoreactive formulations were prepared by dissolving the respective macromers in 0.6 mM Li-TPO in PBS stock solution. The UV exposure time was varied from 1-10 min. (A) Exemplary storage moduli over time, (B) final storage moduli (G'_{max}) of the IPNs, (C) delay time (t_D) of the photopolymerization, (D) slope of the storage modulus over time ($\Delta G'/\Delta t$) during the photopolymerization.

Table S 5: Results of the photorheological analysis of semi-IPNs prepared containing 5 wt% PVA-NB:SS-DT in PI-PBS and 10 wt% Gel in PBS. 0.6 mM Li-TPO were added as photoinitiator. The irradiation time was varied from 1 – 10 min, followed by 9-0 min of "dark" curing afterwards.

	t_D (s)	$\Delta G'/\Delta t$ (Pa s^{-1})	G'_{max} (kPa)
IPN5 – 10 min	3.58 ± 0.27	85.7 ± 2.7	4.99 ± 0.14
IPN5 – 5 min	3.34 ± 0.37	97.5 ± 17.3	4.28 ± 0.11
IPN5 – 1 min	3.03 ± 0.56	91.5 ± 9.2	4.21 ± 1.01

3.2. Optimizing the photopolymerization: variation of the irradiation time

A 385 nm LED lamp was used to irradiate the samples with two different light intensities (18 mW cm^{-2} and 10 mW cm^{-2}). IPN5 (0.6 mM Li-TPO) that was photopolymerized using a 320-500 nm broadband lamp was used as a reference (Figure S 8. Table S5).

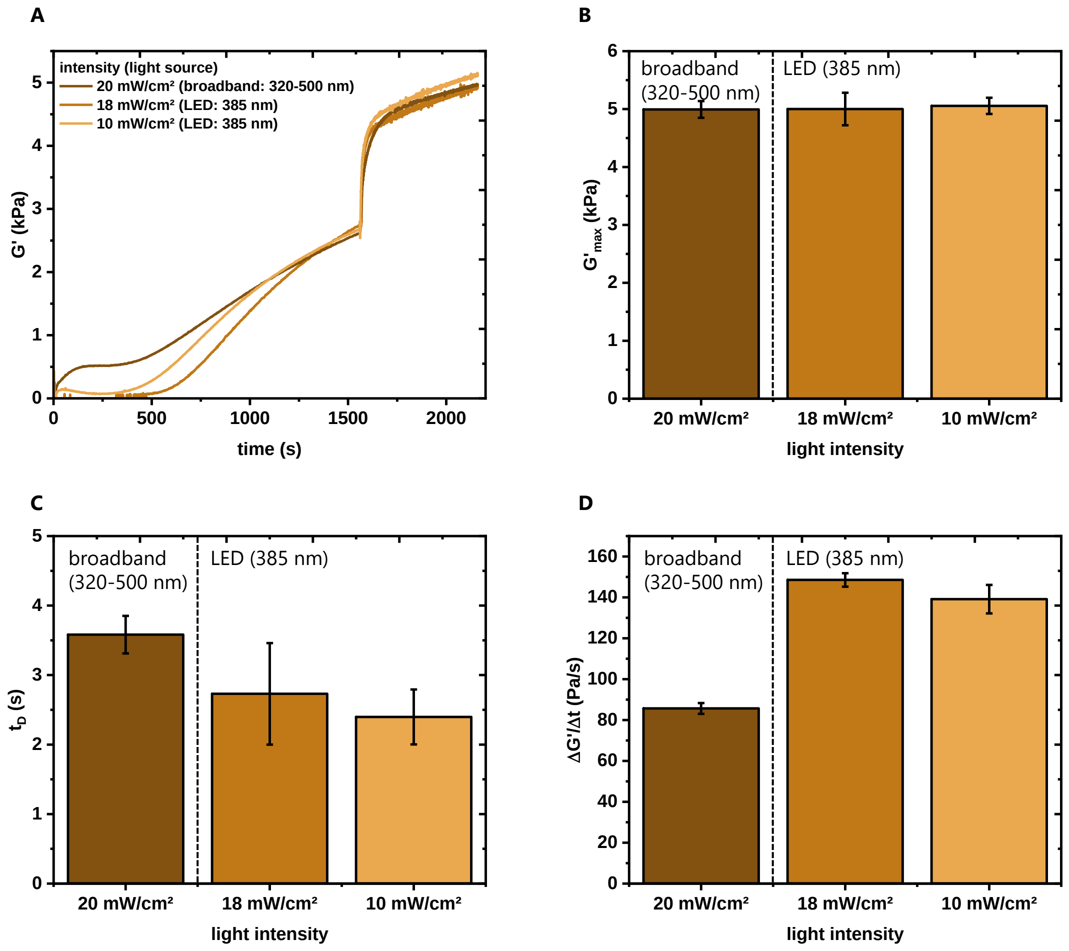

Figure S 8: Results of the photorheological analysis for IPNs composed of 10 wt% gelatin and 5wt% of PVA-NB:SS-DT (IPN5). Photoreactive formulations were prepared by dissolving the respective macromers in 0.6 mM Li-TPO in PBS stock solution. The light source (LED vs. broadband) was varied as well as the light intensity (10-20 mW cm^{-2}). (A) Exemplary storage moduli over time, (B) final storage moduli (G'_{max}) of the IPNs, (C) delay time (t_D) of the photopolymerization, (D) slope of the storage modulus over time ($\Delta G'/\Delta t$) during the photopolymerization.

Table S 6: Results of the photorheological analysis of semi-IPNs prepared containing 5 wt% PVA-NB:SS-DT in PI-PBS and 10 wt% Gel in PI-PBS. 0.6 mM Li-TPO were added as photoinitiator. The irradiation intensity was varied from 10-18 mW cm^{-2}.

		t_D (s)	$\Delta G'/\Delta t$ (Pa s^{-1})	G'_{max} (kPa)
broadband UV	IPN5 – 20 mW/cm²	3.58 ± 0.27	85.7 ± 2.7	4.99 ± 0.14
385 nm LED	IPN5 – 18 mW/cm²	2.73 ± 0.68	148.5 ± 3.3	5.00 ± 0.28
	IPN5 – 10 mW/cm²	2.40 ± 0.42	139.1 ± 6.97	5.06 ± 0.14

3.3. *In-vitro* degradation of semi-IPN hydrogels

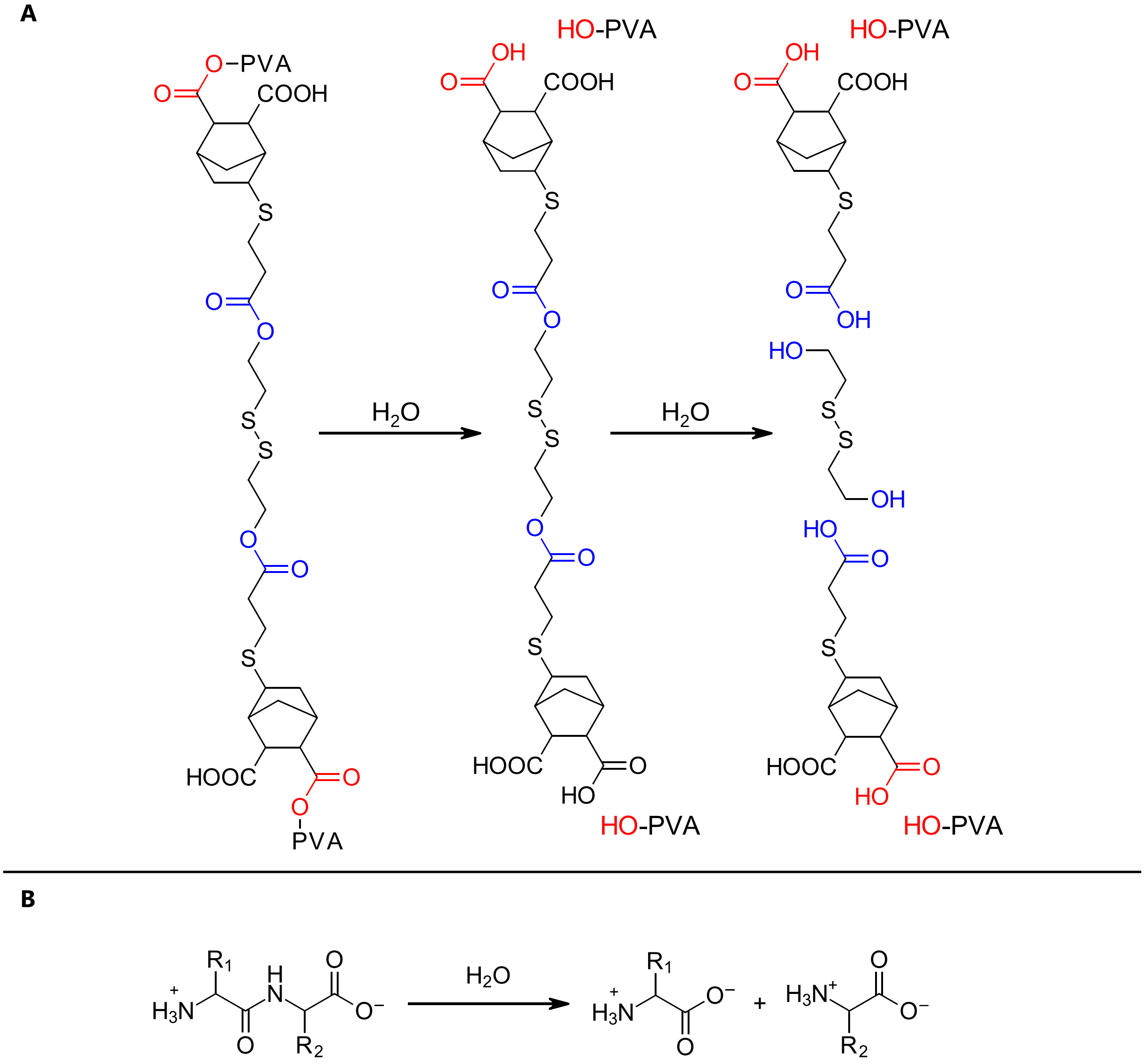

Figure S 9: Proposed hydrolytic degradation of (A) PVA-NB:SS-DT and (B) gelatin macromers.

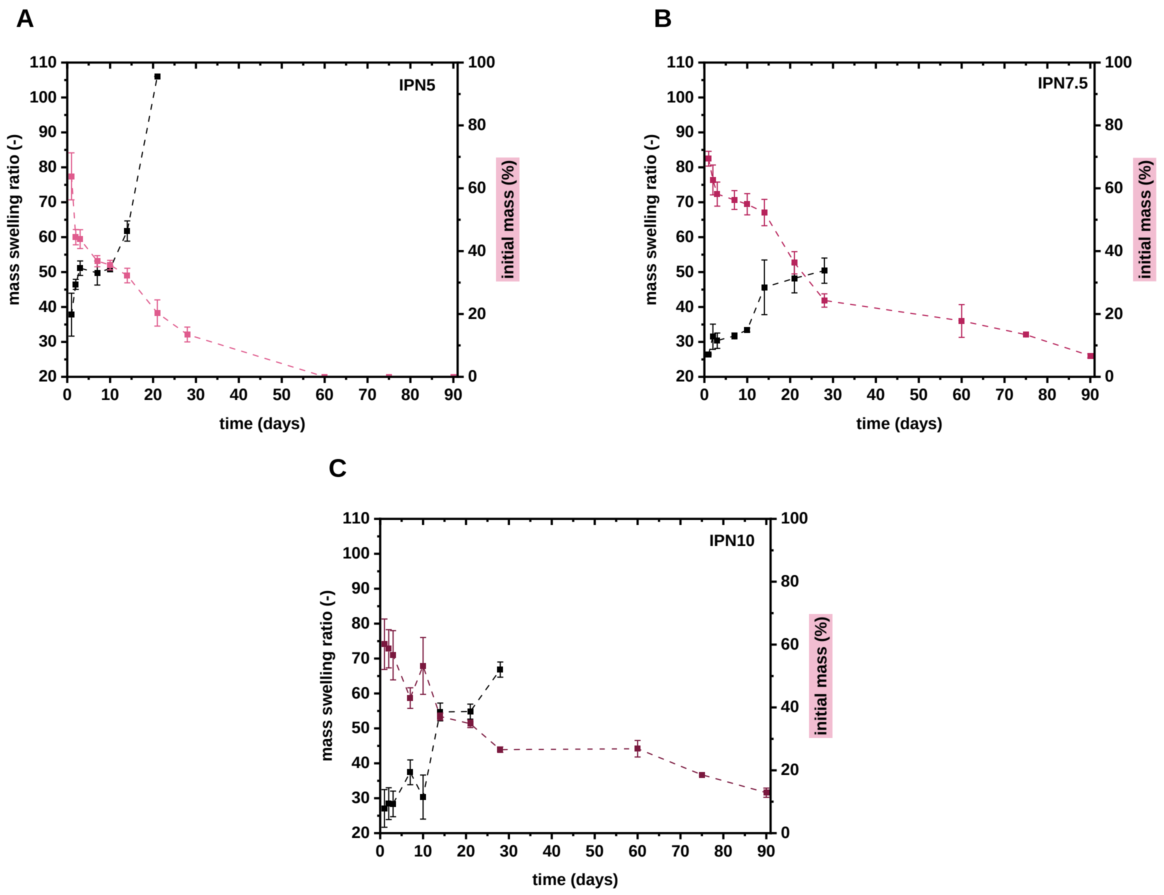

Figure S 10: In-vitro degradability of semi-IPNs at 37 °C containing A) 5 wt% (IPN5), B) 7.5 wt% (IPN 7.5) and C) 10 wt% (IPN10) PVA-NB:SS-DT and 10 wt% gelatin. Mass change of dry mass (initial mass. %) and mass swelling ratio (MSR. -) are plotted over time. MSR was recorded until the samples became too fragile to examine.

*Table S 7: In-vitro degradability of semi-IPNs composed of 5-10 wt% PVA-NB:SS-DT and 10 wt% gelatin: results of the degradation study at 37 °C. *) Samples too fragile to be weighed. **) samples fully degraded in the observed timeperiod.*

	IPN5		IPN7.5		IPN10	
time (days)	MSR (-)	initial mass (%)	MSR (-)	initial mass (%)	MSR (-)	initial mass (%)
1	37.79 ± 6.14	63.79 ± 7.47	26.44 ± 0.21	69.42 ± 2.34	27.07 ± 5.40	60.11 ± 8.04
2	46.49 ± 1.44	44.45 ± 2.44	31.48 ± 3.61	62.65 ± 4.75	28.45 ± 4.55	58.67 ± 6.08
3	51.10 ± 2.08	43.82 ± 3.00	30.34 ± 2.19	58.15 ± 3.83	28.38 ± 3.67	56.60 ± 7.83
7	49.74 ± 3.44	36.79 ± 1.76	31.71 ± 0.84	56.27 ± 2.99	37.42 ± 3.55	42.98 ± 3.28
10	50.99 ± 0.90	35.56 ± 1.52	33.41 ± 0.56	54.91 ± 3.38	30.33 ± 6.32	53.22 ± 9.04
14	61.76 ± 2.90	32.24 ± 2.31	45.63 ± 7.82	52.28 ± 4.19	54.70 ± 2.54	37.10 ± 1.13
21	106.02 ± 0.03	20.31 ± 4.16	48.19 ± 4.14	36.30 ± 3.55	54.80 ± 2.14	34.80 ± 1.21
28	*	13.46 ± 2.36	50.40 ± 3.61	36.29 ± 2.12	66.84 ± 2.17	26.56 ± 0.87
60	*	0**	*	27.77 ± 5.21	*	26.86 ± 2.64
75	*	0**	*	13.4 ± 0.20	*	18.56 ± 0.36
90	*	0**	*	6.67 ± 0.65	*	12.87 ± 1.46

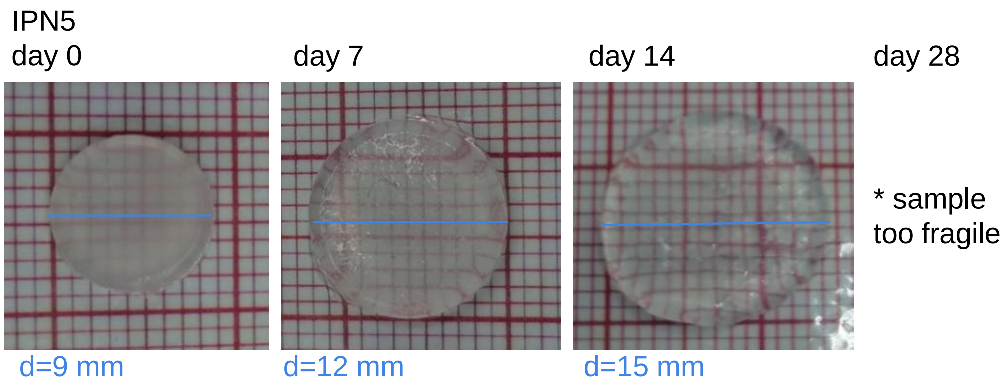

Figure S 11: Photographs of IPN5 at different degradation time points (pH = 7.4. 37 °C). From day 28 on. the samples became too fragile and were directly subjected to lyophilization.

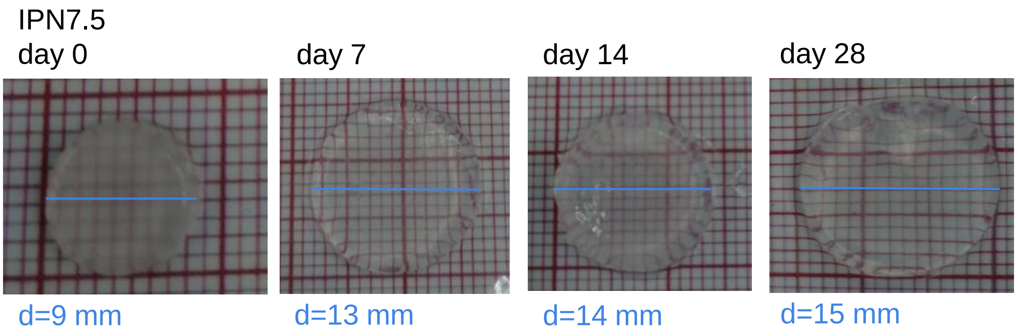

Figure S 12: Photographs of IPN7.5 at different degradation time points (pH = 7.4. 37 °C). From day 60 on. the samples became too fragile and were directly subjected to lyophilization.

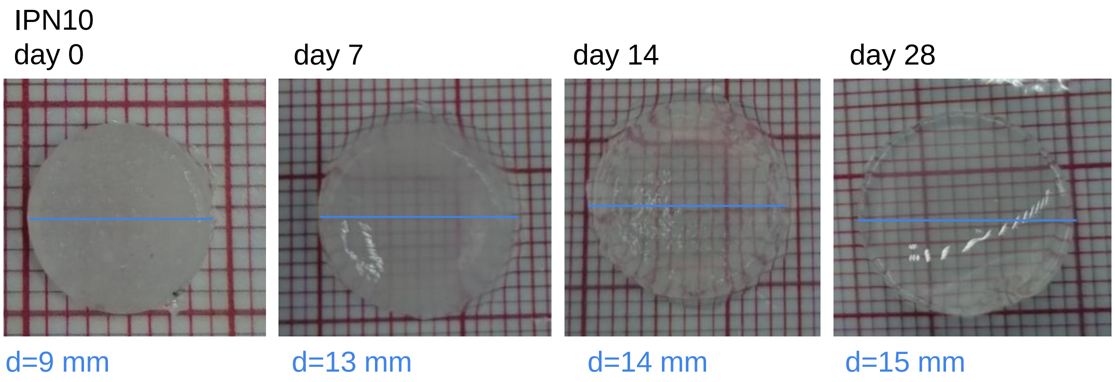

Figure S 13: Photographs of IPN10 at different degradation time points (pH = 7.4. 37 °C). From day 60 on. the samples became too fragile and were directly subjected to lyophilization

4. NETWORK STABILIZATION BY HYBRID PVA- AND GEL-NB HYDROGELS

4.1. *In-situ* photorheology measurements of Gel-NB:SS-DT (10 wt%)

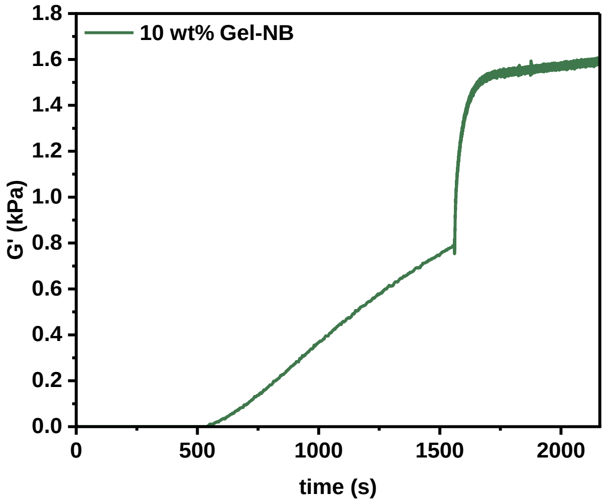

Figure S 14: Representative storage moduli over time for a 10 wt% Gel-NB:SS-DT formulation. 0.6 mM Li-TPO were used as initiator.

Table S 8: Results of the in-situ photorheology of 10 wt% Gel-NB:SS-DT. 0.6 mM Li-TPO were used as PI.

	$G'_{phys. Gel}$ (kPa)	t_D (s)	$\Delta G'\ \Delta t^{-1}$ (Pa s^{-1})	G'_{max} (kPa)
Gel-NB:SS-DT	0.81 ± 0.06	1.23 ± 0.26	35.3 ± 12.8	1.74 ± 0.22

Table S 9: Results of the photorheological analysis of hydrogels prepared from 5-10 wt% PVA-NB:SS-DT in PI-PBS and 10 wt% Gel-NB:SS-DT in PI-PBS. 0.6 mM Li-TPO in PBS were added as photoinitiator.

	t_D (s)	$\Delta G'/\Delta t$ (Pa s^{-1})	G'_{max} (kPa)
5 wt% PVA-NB + 10 wt% Gel-NB	1.52 ± 0.03	167 ± 30	5.97 ± 0.29
7.5 wt% PVA-NB + 10 wt% Gel-NB	1.78 ± 0.14	302 ± 75	10.5 ± 1.0
10 wt% PVA-NB + 10 wt% Gel-NB	0.29 ± 0.09	655 ± 56	16.4 ± 1.0

4.2. *In-vitro* degradation of hybrid PVA- and Gel-NB hydrogels

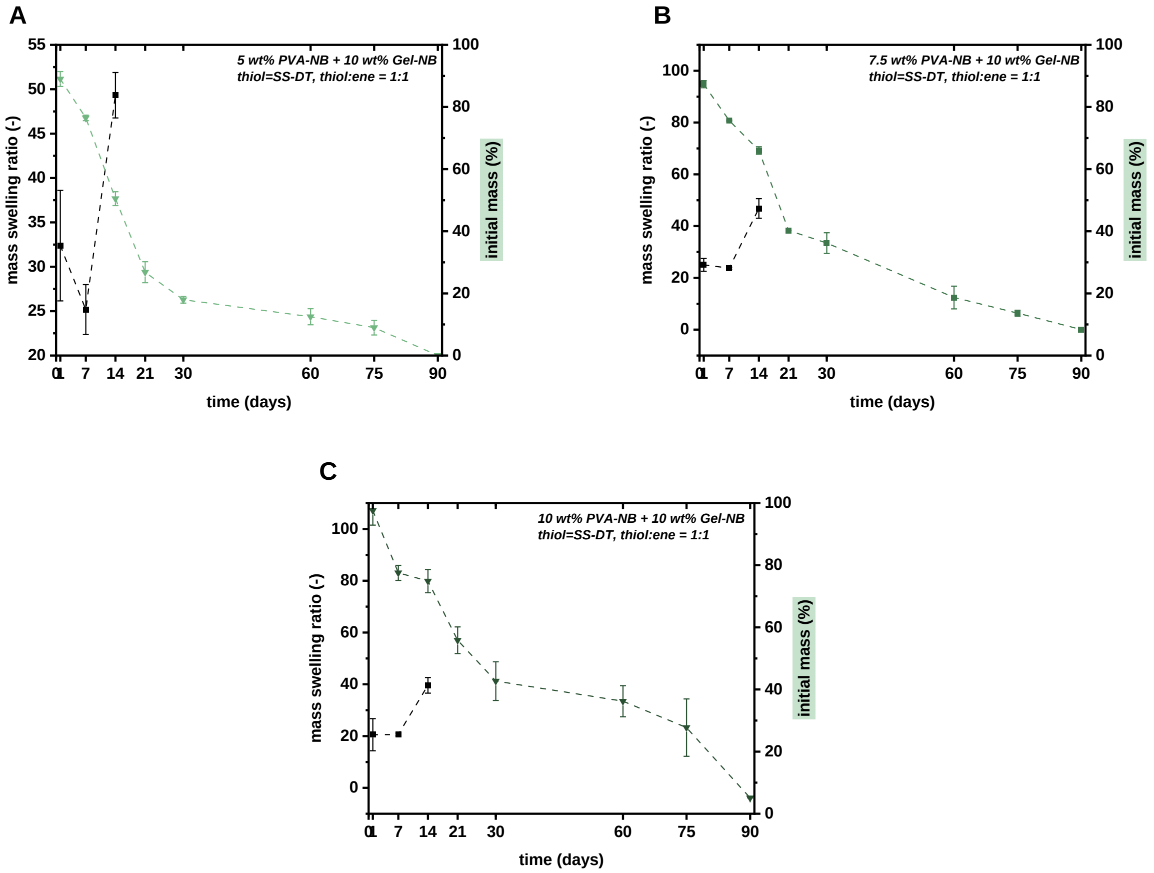

Figure S 15: In-vitro degradability of hybrid materials at 37 °C containing A) 5 wt%, B) 7.5 wt% and C) 10 wt% PVA-NB:SS-DT and 10 wt% Gel-NB:SS-DT. Mass change of dry mass (initial mass, %) and mass swelling ratio (MSR, -) are plotted over time. MSR was recorded until the samples became too fragile to examine.

*Table S 10: In-vitro degradability of hydrogels composed of 5-10 wt% PVA-NB:SS-DT and 10 wt% Gel-NB:SS-DT: results of the degradation study at 37 °C. *) Samples too fragile to be weighed, **) samples fully degraded in the observed timeperiod.*

	5 wt% PVA-NB + 10 wt% Gel-NB		7.5 wt% PVA-NB + 10 wt% Gel-NB		10 wt% PVA-NB + 10 wt% Gel-NB	
time (days)	MSR (-)	initial mass (%)	MSR (-)	initial mass (%)	MSR (-)	initial mass (%)
1	32.4 ± 6.22	92.0 ± 2.4	25.0 ± 2.5	94.8 ± 1.4	20.5 ± 6.2	97.6 ± 4.7
7	25.2 ± 2.8	76.5 ± 0.9	23.7 ± 0.4	80.8 ± 0.2	20.6 ± 0.5	77.5 ± 2.4
14	49.3 ± 2.6	50.5 ± 2.2	46.8 ± 3.8	69.2 ± 1.4	39.6 ± 3.0	74.9 ± 3.7
21	*	26.8 ± 3.4	*	38.3 ± 0.1	*	55.9 ± 4.3
28	*	17.9 ± 1.1	*	33.4 ± 4.0	*	42.7 ± 6.2
60	*	12.5 ± 2.6	*	12.4 ± 4.4	*	36.2 ± 5.0
75	*	9.0 ± 2.3	*	6.4 ± 1.1	*	27.7 ± 9.2
90	*	0**	*	0**	*	5.1 ± 0.1

5. TWO-PHOTON MICROPATTERNING OF DISULFIDE-BEARING HYDROGELS

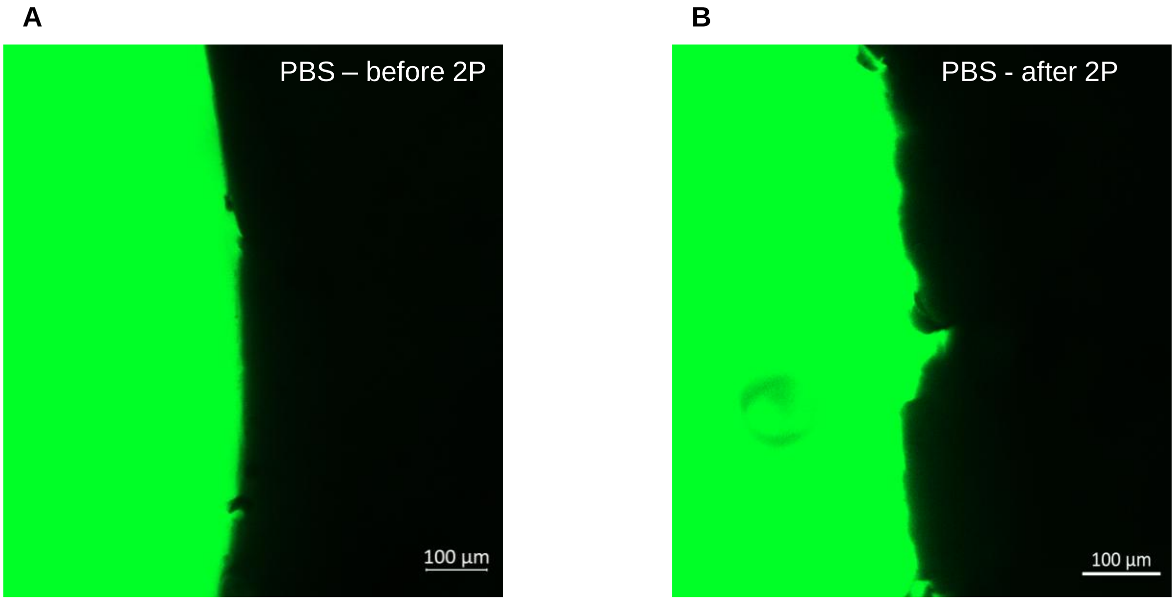

Figure S 16: IPN5 swollen in PBS (A) before and (B) after two-photon micropatterning. No fluorescence of FITC200 (green) was observed within the bulk hydrogels (black) after laser exposure.

5.1. Preliminary two-photon micropatterning of IPN5

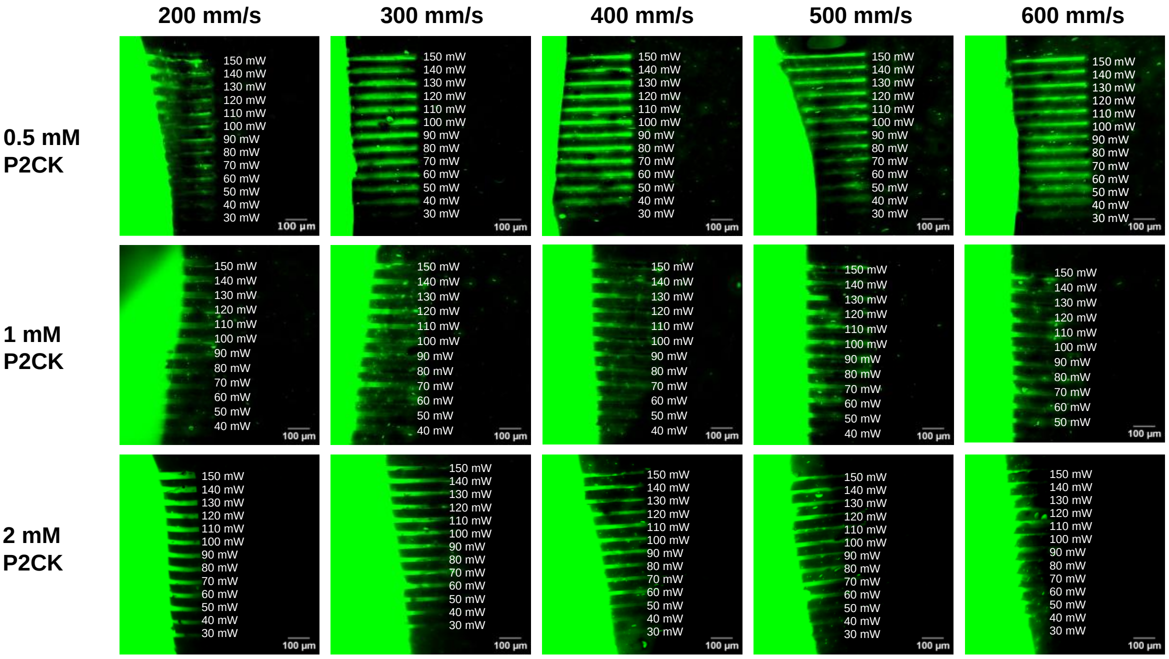

Figure S 17: Microchannels were fabricated by two-photon degradation of disulfide based IPN5 in the presence of two-photon initiator P2CK (0.5-2.0 mM). Individual x,y-planes were either scanned with variable laser power of 30-150 mW or scanning speeds of 200-600 mm s^{-1}. Thereafter, hydrogels were soaked in a solution of high molecular weight fluorescent dextran (FITC2000) for 18 h and microchannels were visualized by confocal microscopy. Scale bar 100 µm.

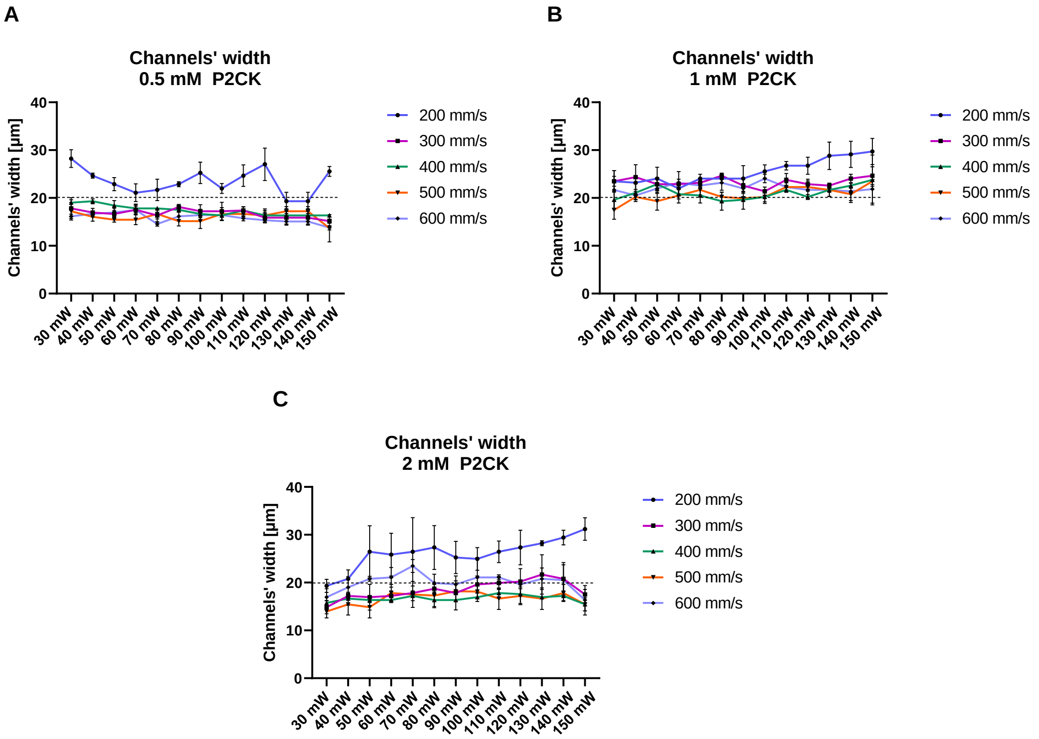

Figure S 18: Calculated channel widths of IPN5 after 2P-micropatterning using 0.5 (A), 1.0 (B) or 2.0 (C) mM P2CK. Scanning speed (200-600 mm/s) and laser power (30-150 mW) were varied. The theoretical channel width of 20 µm is indicated as dotted line. The channels were divided into three equal parts wherefrom the average channel width (+standard deviation) was calculated.

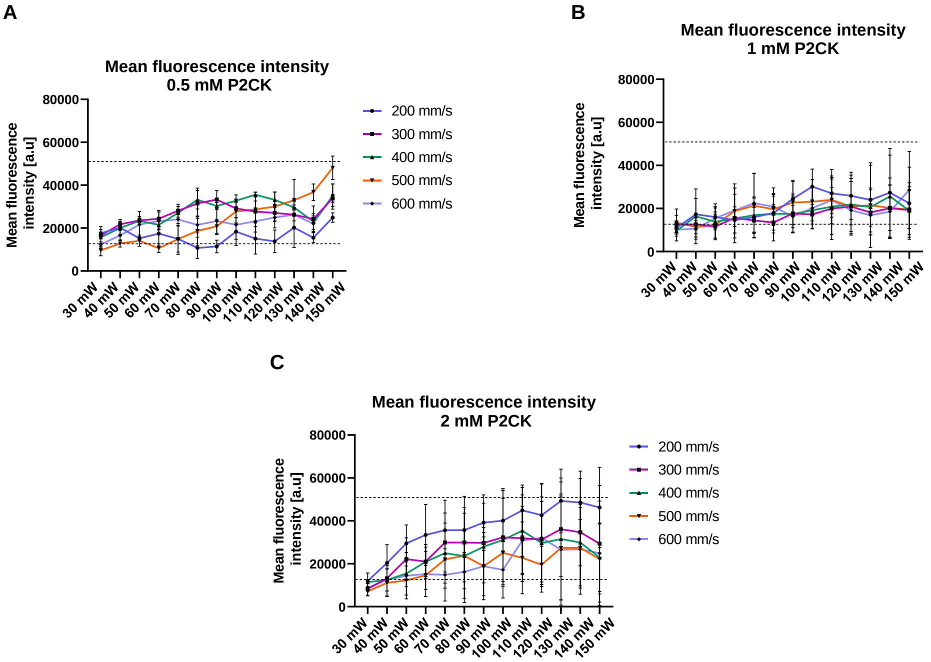

Figure S 19: Mean fluorescence intensities of IPN5 after 2P-micropatterning using 0.5 (A), 1.0 (B) or 2.0 (C) mM P2CK. Scanning speed (200-600 mm/s) and laser power (30-150 mW) were varied. The upper and lower dashed lines represent the reference fluorescence intensity (mean fluorescence intensity surrounding the hydrogel droplet) and the background fluorescence signal (in the non-micropatterned hydrogel, resulting from partial dye diffusion over time), respectively. The channels were divided into three equal parts wherefrom the average channel width (+standard deviation) was calculated.

5.2. Two-photon micropatterning of IPN5 and hybrid gels, imaging on day 1

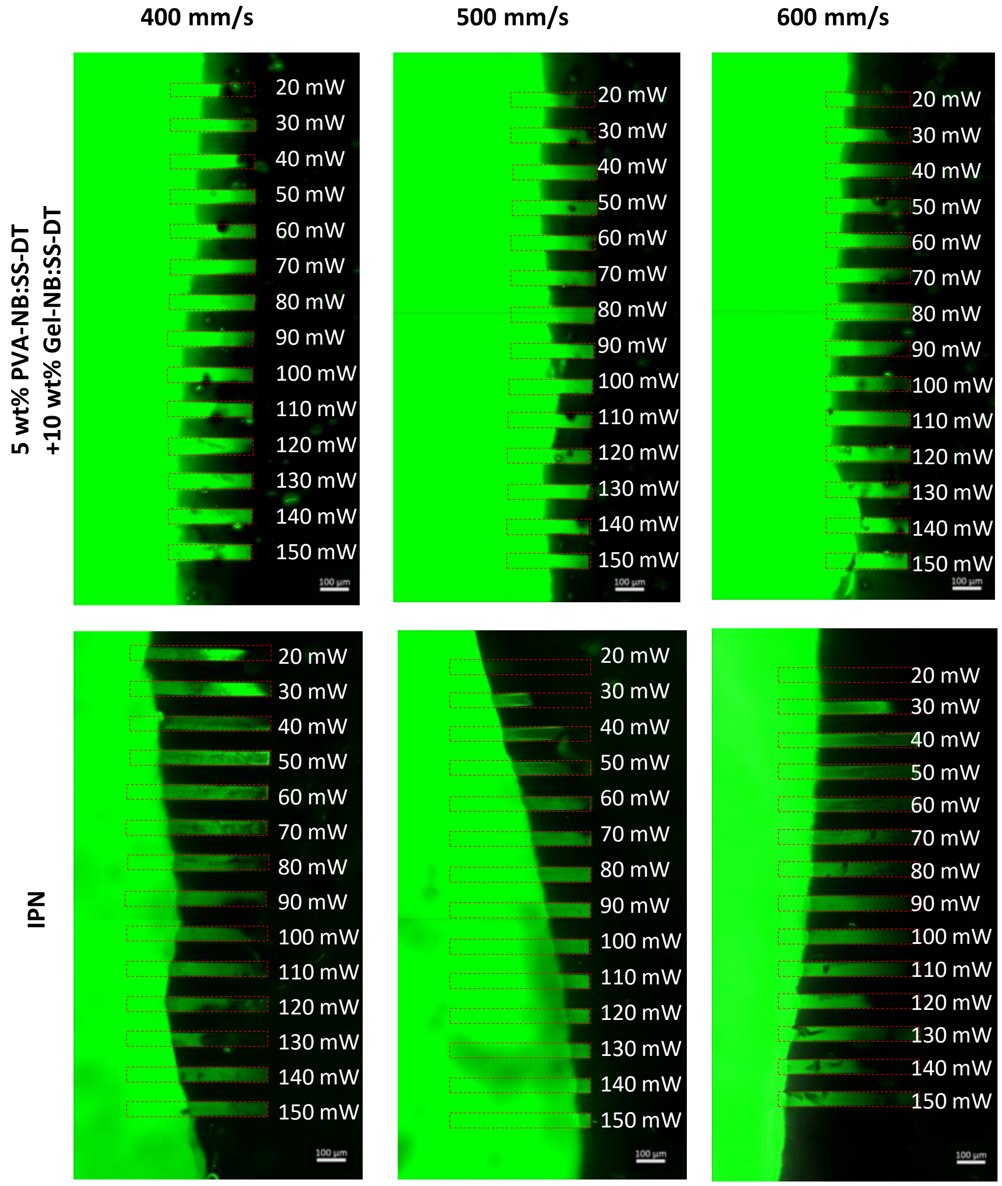

Figure S 20: Microchannels were fabricated by two-photon degradation of disulfide based (A) IPN5 and (B) disulfide-containing hybrid hydrogels (5 wt% PVA-NB:SS-DT+10 wt% Gel-NB:SS-DT) in the presence of two-photon initiator P2CK (0.5 mM). Individual x,y-planes were either scanned with variable laser power of 20-150 mW or scanning speeds of 400-600 mm s^{-1}. Thereafter, hydrogels were soaked in a solution of high molecular weight fluorescent dextran (FITC2000) for 18 h and microchannels were visualized by confocal microscopy. Scale bar 100 µm. Channels are visualized by dotted outlines (red).

5.3. Two-photon micropatterning of IPN5 and hybrid gels, imaging on day 7

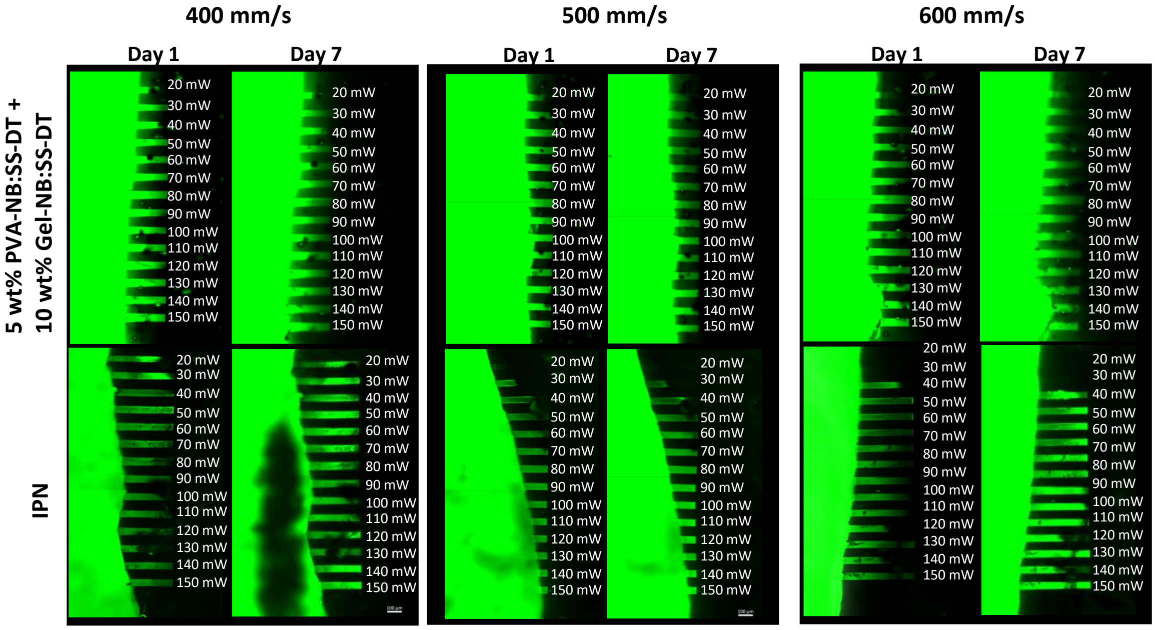

Figure S 21: Microchannels were fabricated by two-photon degradation of disulfide based IPN5 and disulfide-containing hybrid hydrogels (5 wt% PVA-NB:SS-DT+10 wt% Gel-NB:SS-DT) in the presence of two-photon initiator P2CK (0.5 mM). Individual x,y-planes were either scanned with variable laser power of 20-150 mW or scanning speeds of 400-600 mm s^{-1}. Thereafter, hydrogels were soaked in a solution of high molecular weight fluorescent dextran (FITC2000) for 18 h and microchannels were visualized by confocal microscopy. The visualization was repeated after 7 days. Scale bar 100 µm.

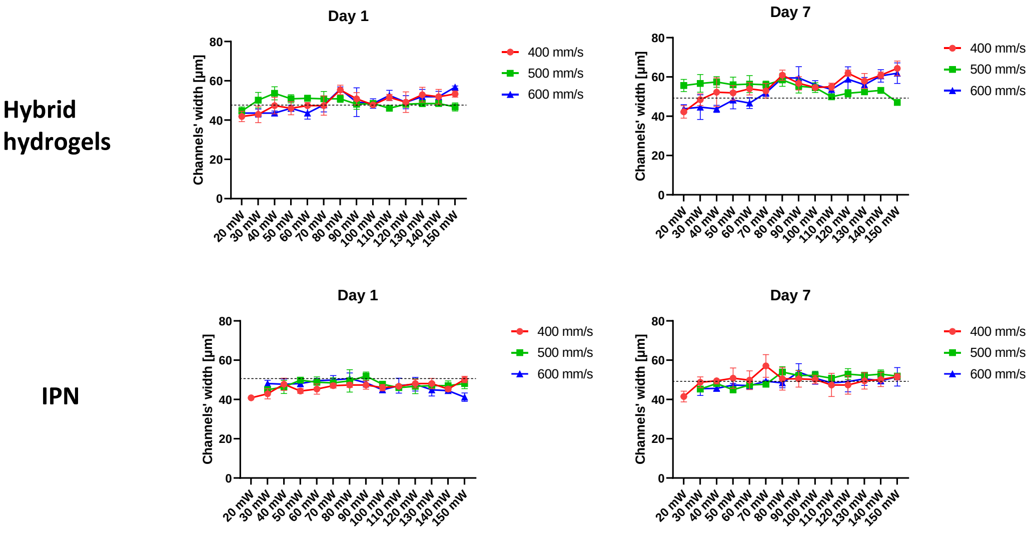

Figure S 22: Calculated channel widths of IPN5 and hybrid gels after 2P-micropatterning after day 1 and day 7. Scanning speed (400-600 mm/s) and laser power (20-150 mW) were varied. The theoretical channel width of 50 µm is indicated as dotted line. The channels were divided into three equal parts wherefrom the average channel width (+standard deviation) was calculated.

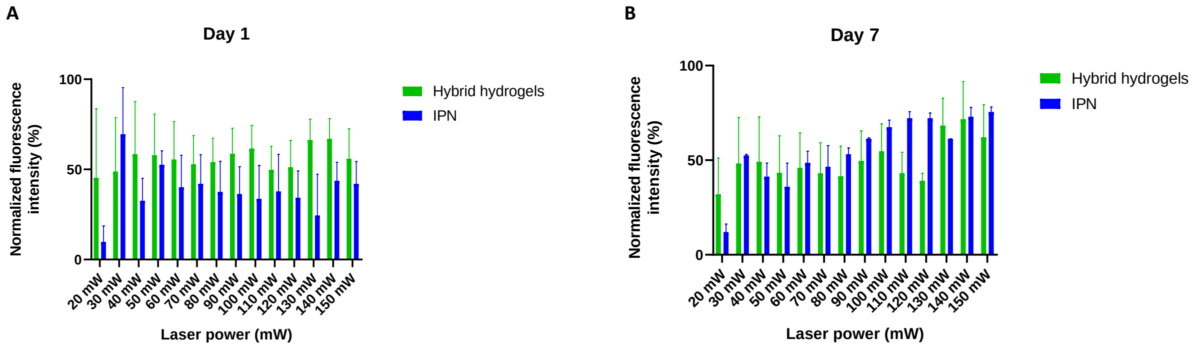

Figure S 23: Normalized fluorescence intensities of IPN5 (blue) and hybrid gels (green) after 2P-micropatterning. Fluorescence was measured at (A) day 1 or (B) day 7. A fixed scanning speed of 500 mm/s was used and the laser power (20-150 mW) were varied. The channels were divided into three equal parts wherefrom the average channel width (+standard deviation) was calculated.

6. EFFECTIVE SWELLING DATA

Effective swelling data was calculated from the initial wet weight ($m_{t=0,wet}$) and the swollen weight ($m_{t=x,swollen}$) according to Equation S1:

$$effective\ swelling\ (-) = \frac{m_{t=x,swollen}}{m_{t=0,wet}} \tag{S1}$$

Table S 11: Effective swelling data for all hydrogels during the in-vitro degradation study under physiological conditions.

	IPNs			x wt% PVA-NB:SS-DT			x wt% PVA-NB:SS-DT + 10 wt% Gel-NB:SS-DT		
day	IPN5	IPN7.5	IPN10	5	7.5	10	5	7.5	10
1	3.64 ± 0.19	3.23 ± 0.10	3.20 ± 0.23	4.59 ± 0.01	4.76 ± 0.17	4.28 ± 0.04	2.83 ± 0.42	3,61 ± 0.35	4,34 ± 0.57
2	3.16 ± 0.11	3.45 ± 0.24	3.30 ± 0.25	4.75 ± 0.37	5.10 ± 0.37	4.73 ± 0.09	-	.	-
3	3.43 ± 0.28	3.09 ± 0.16	3.18 ± 0.24	4.89 ± 0.10	4.23 ± 1.44	4.55 ± 0.12	-	.	-
7	2.80 ± 0.08	3.14 ± 0.24	3.20 ± 0.16	5.02 ± 0.02	4.76 ± 0.10	4.83 ± 0.20	2.46 ± 0.04	3.00 ± 0.05	2.90 ± 0.31
10	2.77 ± 0.15	3.23 ± 0.15	3.15 ± 0.07	4.94 ± 0.12	4.95 ± 0.11	4.85 ± 0.10	-	-	-
14	3.04 ± 0.10	4.16 ± 0.45	4.06 ± 0.25	5.13 ± 0.08	5.36 ± 0.79	5.03 ± 0.22	2.50 ± 0.11	3,28 ± 0.71	2.12 ± 1.90
21	2.20 ± 2.0	3.06 ± 0.17	3.47 ± 0.38	5.01 ± 012	4.28 ± 1.20	4.97 ± 0.08	**	**	**
28	**	3.21 ± 0.18	3.55 ± 0.16	**	5.53 ± 0.59	4.96 ± 0.05	**	**	**
60	**	**	**	**	**	**	**	**	**
75	**	**	**	**	**	**	**	**	**
90	**	**	**	**	**	**	**	**	**
